# Supplementary material for: Identifying T-cell clubs by embracing the local harmony between TCR and gene expressions
Source: Mol Syst Biol. 2024 Nov 4;20(12):1329–45. doi: 10.1038/s44320-024-00070-5 (PMC11612385; doi:10.1038/s44320-024-00070-5)
Supplement: Supplementary file 1 — Appendix [file 44320_2024_70_MOESM1_ESM.pdf]

Appendix Supplementary Materials for

Identifying T-cell clubs by embracing the local harmony between TCR and gene expressions

Yiping Zou *et al.*

This PDF file includes:

Appendix Table S1..pages 2-3
Appendix Figures S1 to S36..pages 4-29
Appendix Supplementary Methods..pages 30-36

Data Type	Full Name	Accession Number	URL	Main Purpose	Samples
scRNA + DNA-encoded peptide-HLA tetramers + scTCR	Gene Expression Omnibus	GSE188429	https://www.ncbi.nlm.nih.gov/geo/query/acc.cgi?acc=GSE188429	Assess the performance of clustering T cells	EXP20000654_Sample_02<br>EXP20000654_Sample_03<br>EXP20000654_Sample_04<br>EXP20000654_Sample_05<br>EXP20000654_Sample_06<br>EXP20000683_Sample_07<br>EXP20000683_Sample_08<br>EXP20000722_Sample_01<br>EXP20000722_Sample_03<br>EXP20000722_Sample_04<br>EXP20000722_Sample_05<br>EXP20000722_Sample_06<br>EXP20000722_Sample_07<br>EXP20000768_Sample_01<br>EXP20000768_Sample_02<br>EXP20000768_Sample_03<br>EXP20000768_Sample_04<br>EXP20000780_Sample_02<br>EXP20000780_Sample_03<br>EXP20000854_Sample_01<br>EXP20000854_Sample_02<br>EXP20000854_Sample_03<br>EXP20000854_Sample_04<br>EXP20000854_Sample_05<br>EXP20000854_Sample_06<br>EXP20000910_Sample_01<br>EXP20000910_Sample_02<br>EXP20000910_Sample_03<br>EXP20000910_Sample_04<br>EXP20000910_Sample_05<br>EXP20000910_Sample_06<br>EXP20000910_Sample_07<br>EXP20000910_Sample_08<br>EXP20000910_Sample_09<br>EXP20000910_Sample_10<br>EXP20000910_Sample_11<br>EXP20000910_Sample_12<br>EXP20001003_Sample_02<br>EXP20001003_Sample_04<br>EXP20001003_Sample_05<br>EXP20001003_Sample_06<br>EXP20001003_Sample_07<br>EXP20001003_Sample_08<br>EXP20001095_Sample_01<br>EXP20001095_Sample_02<br>EXP20001095_Sample_03<br>EXP20001095_Sample_04<br>EXP20001114_Sample_01<br>EXP20001114_Sample_02<br>EXP20001114_Sample_03<br>EXP20001114_Sample_04<br>EXP20001114_Sample_05<br>EXP20001114_Sample_06<br>EXP20001114_Sample_07<br>EXP20001114_Sample_08

scRNA + cell surface protein+ scTCR	10x Genomics Datasets	CD8+ T cells of Healthy Donor	https://www.10xgenomics.com/resources/datasets?query=CD8	Saliency Map; Generalization test	Donor 1-4.
scRNA + scTCR	Gene Expression Omnibus	GSE123813	https://www.ncbi.nlm.nih.gov/geo/query/acc.cgi?acc=GSE123813	Analysis of BCC patients	su001, su002, su003, su004, su005, su006, su007, su008, su009, su010_BCC, su012.
scRNA + scTCR	Gene Expression Omnibus	GSE165080	https://www.ncbi.nlm.nih.gov/geo/query/acc.cgi?acc=GSE165080	Analysis of COVID-19 patients with different symptoms	COV144, COV146, COV147, COV154, COV155, COV048, COV072, COV074, COV076, COV086, COV087, COV089, COV091, COV098, COV101, COV102, COV156, COV157, COV011, COV078, COV012, COV007, COV009, COV013, COV016, COV021, COV029, COV047, COV057, COV037, COV045, COV055, COV119, COV126, COV158, COV159, COV161, COV163, COV164, COV165, COV166, NC1, NC05, NC31, NC36, NC07, NC20, NC21, NC23, NC39, NC58, NC60.
scRNA + scTCR	Gene Expression Omnibus	GSE201425	https://www.ncbi.nlm.nih.gov/geo/query/acc.cgi?acc=GSE201425	Analysis of CCA patients	230093, 230498, 357818, 230723, 231260
scRNA + scTCR	Gene Expression Omnibus	GSE114724	https://www.ncbi.nlm.nih.gov/geo/query/acc.cgi?acc=GSE114724	Appendix Figure S1, S25, S31, S32	BC09_TUMOR1, BC09_TUMOR2, BC10_TUMOR1, BC11_TUMOR1, BC11_TUMOR2
scRNA + scTCR	Gene Expression Omnibus	GSE121636	https://www.ncbi.nlm.nih.gov/geo/query/acc.cgi?acc=GSE121636	Appendix Figure S1, S25, S31, S32	GU0700_T, GU0715_T, GU0744_T, GU0700_P, GU0715_P, GU0744_P.

Appendix Table S1. Data availability

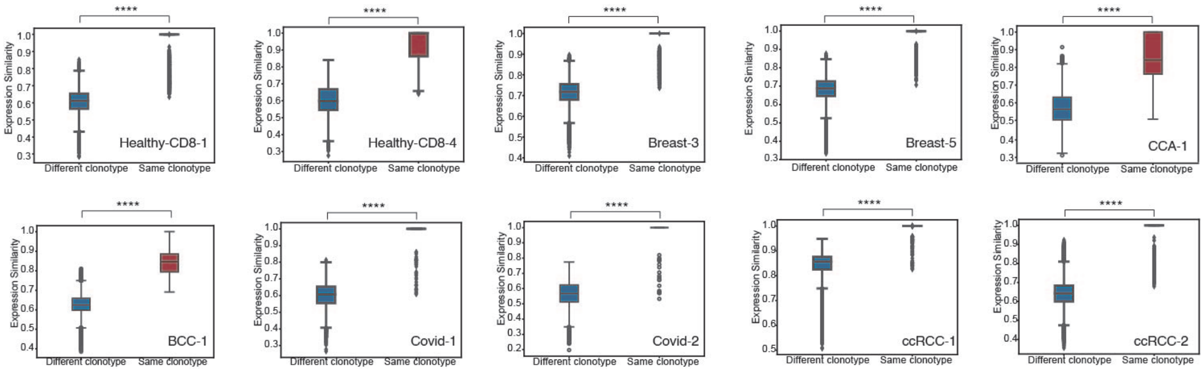

Appendix Figure S1. Expression similarity between T cells belonging to the same clone versus different clonotypes. We examined the RNA expression similarity between pairs of T cells that belong to the same clone versus those that do not. We calculated the dot product of the normalized RNA vectors as a measure of their RNA distances or similarity. Given that the vectors are normalized, the dot product is equivalent to the Euclidean distance. In other words, a larger dot product indicates a larger similarity, or a smaller distance. In the figure, we show the results for ten samples from various datasets. All of the samples have passed one-sided Mann-Whitney's U-test. $^{****}P < 10^{-4}$.

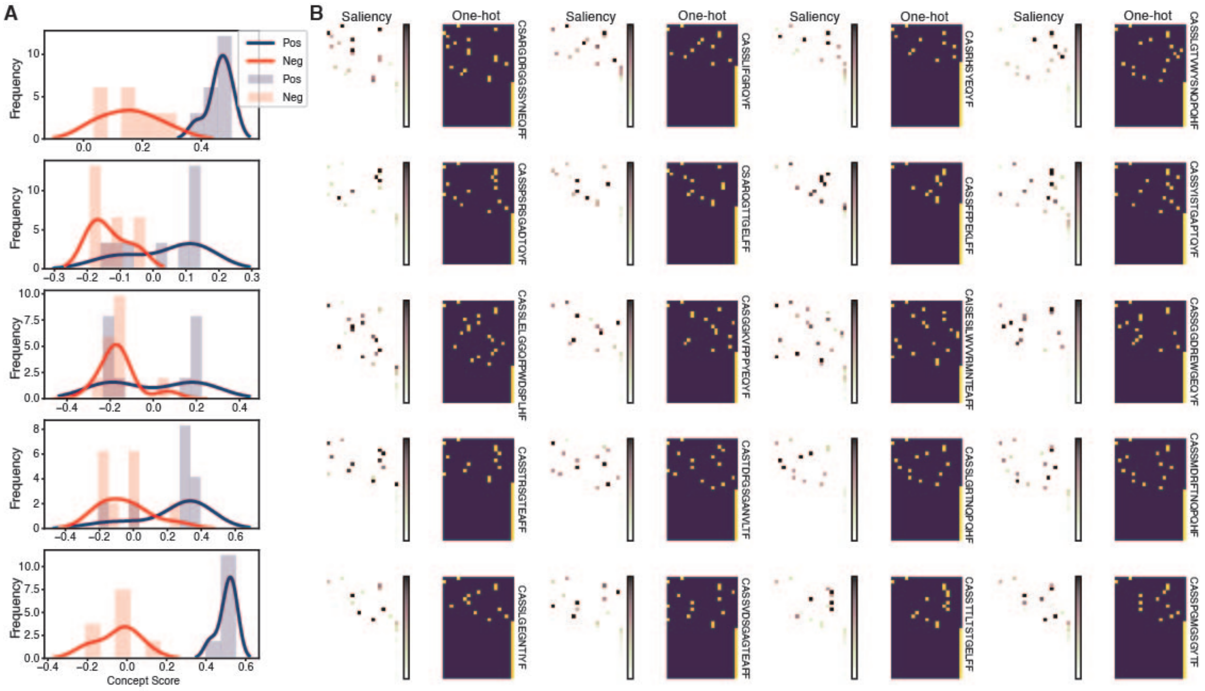

Appendix Figure S2. Saliency maps and density histograms for clubs. Each row represents a density histogram for a particular club and its corresponding saliency maps for the TCRs within the club. All clubs presented different prominent epitopes. The density histograms (**A**) illustrate the concept scores distribution for the club of interest (in blue) and the other clubs from the same sample (in red). The heatmap (**B**) displays the saliency map and the one-hot embedding for TCRs. Only four TCRs with the top saliency scores in every club are shown. The dark-background heatmap indicates the one-hot embedding (see Appendix Supplementary Methods) for each TCR, while the white-background heatmap represents the saliency of each amino acid in the TCR. The colorbar denotes the saliency level, with deeper colors indicating higher importance.

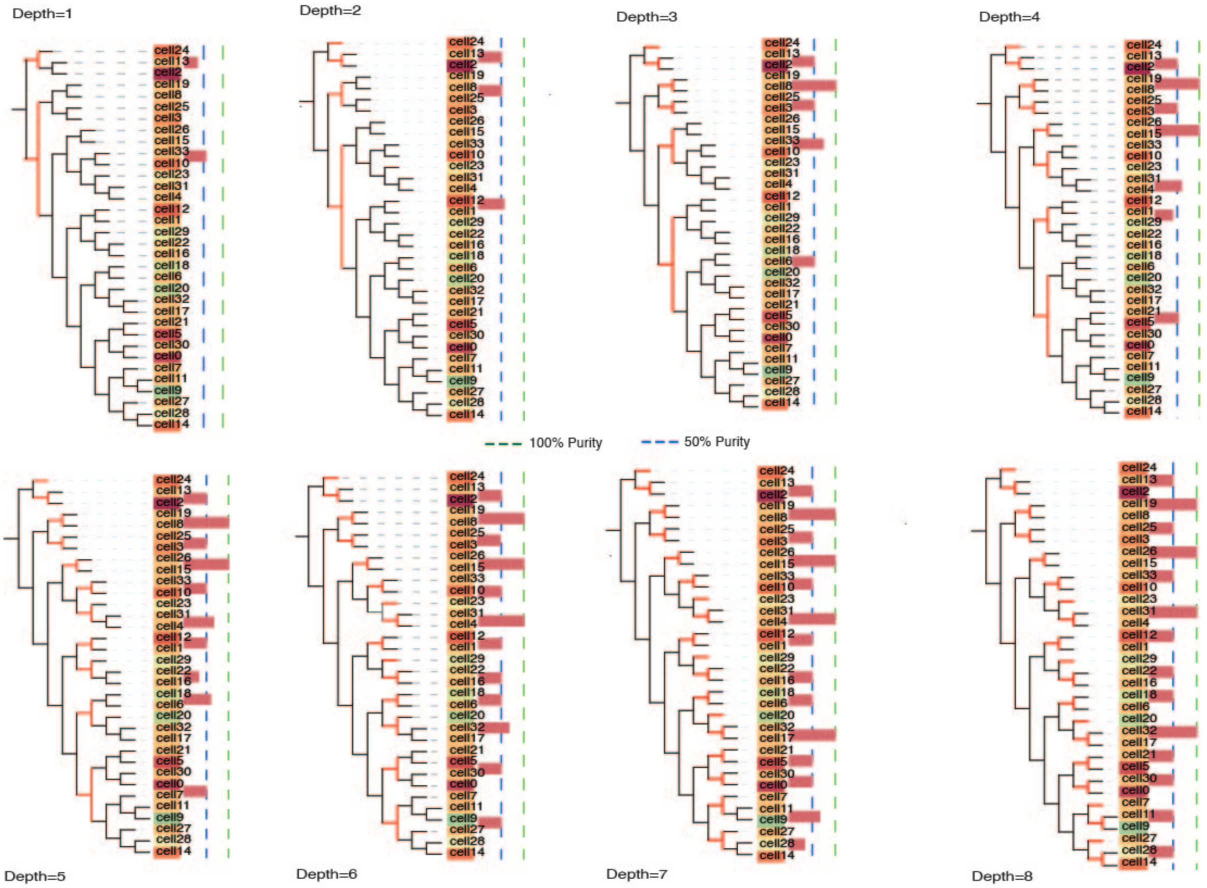

Appendix Figure S3. Clustering performance at different tree depths. The tree-like structure generated by TCRclub was cut at various depths. T cells are color-coded according to their verified pMHC specificity, with identical colors indicating T cells that recognize the same pMHC.

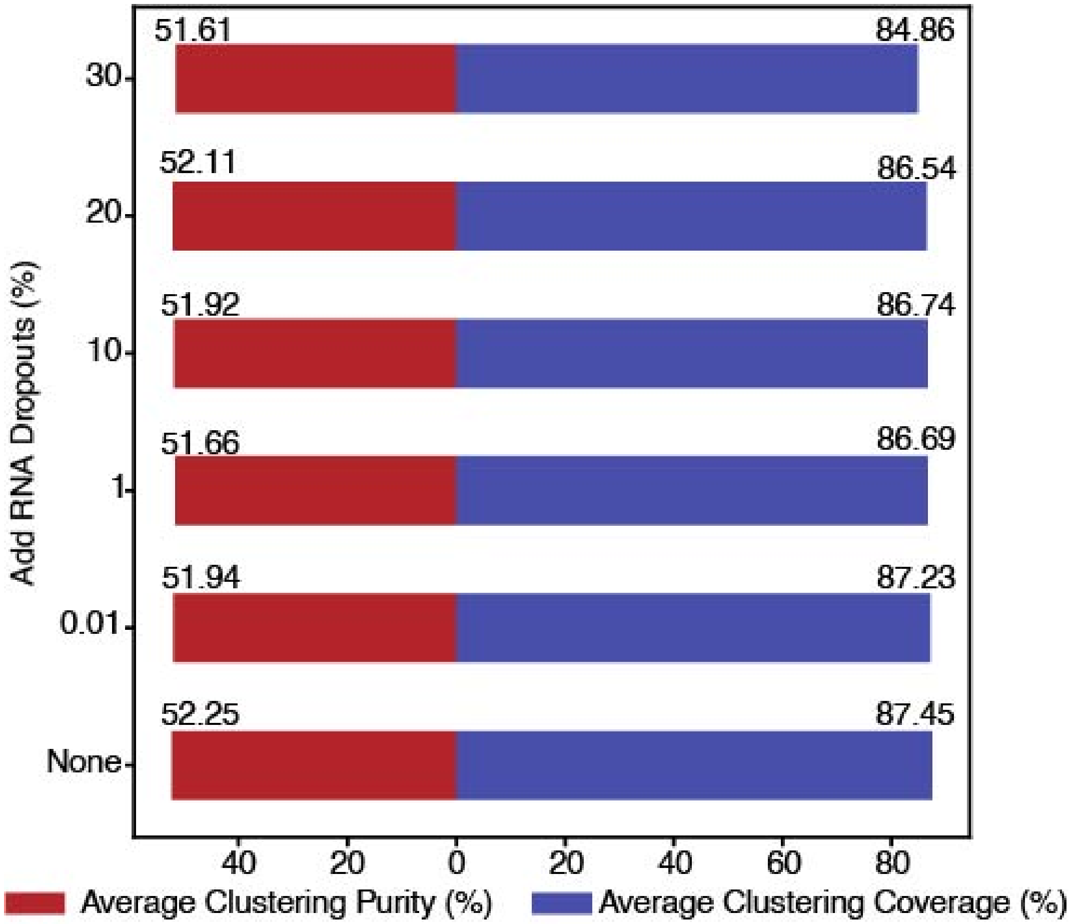

Appendix Figure S4. Effect of RNA dropouts on TCRclub clustering performance. To assess the impact of RNA dropouts on clustering performance, we introduced additional dropouts into the raw RNA count data on the dataset. We calculated the number of non-zero entries in the raw count matrix and randomly set a specified percentage of these non-zero entries to zero. For example, “0.01" indicates that 0.01% of the non-zero entries were randomly set to zero. “None" indicates that no additional dropouts were added. After introducing the dropouts, the RNA gene expression matrix was processed according to the steps outlined in the “Preprocessing for scRNA Profile" section. We compared the average clustering purity and clustering coverage of the dataset for the different ratios of dropouts.

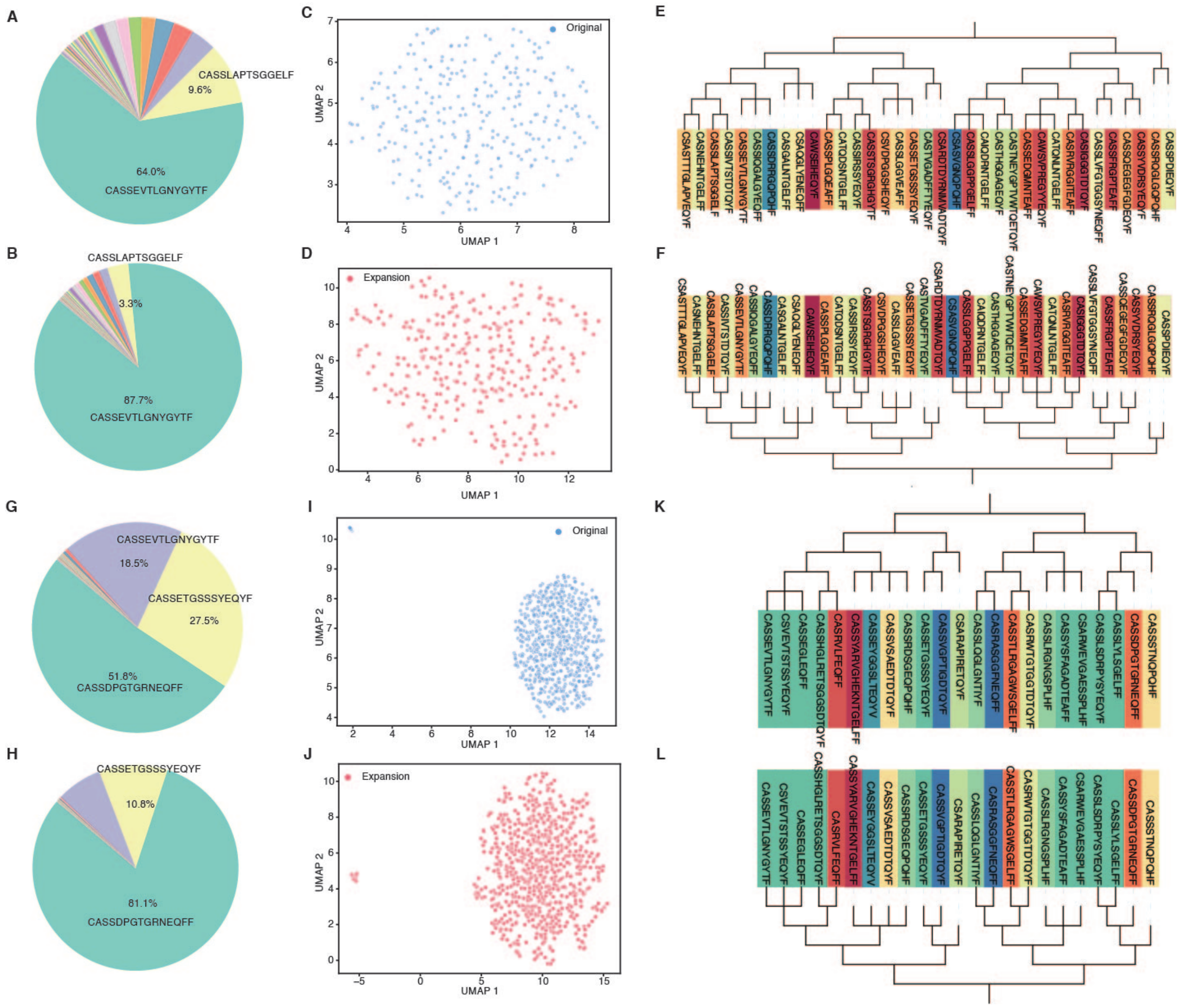

Appendix Figure S5. Impact of Extreme Clonal Expansion on Clustering Results of TCRclub. Two representative examples are presented to illustrate the effect of extreme clonal expansion on TCRclub clustering results. **A**, Original T-cell clone distribution. **B**, T-cell clone distribution after the expansion of CASSEVTLGNYGYTF. **C**, Original RNA expression of T-cell clone CASSEVTLGNYGYTF. **D**, RNA expression of T-cell clone CASSEVTLGNYGYTF after expansion. **E**, Clustering result before clonal expansion. **F**, Clustering result after clonal expansion. **G**, Original T-cell clone distribution. **H**, T-cell clone distribution after the expansion of CASSDPGTGRNEQFF. **C**, Original RNA expression of T-cell clone CASSDPGTGRNEQFF. **D**, RNA expression of T-cell clone CASSDPGTGRNEQFF after expansion. **E**, Clustering result before clonal expansion. **F**, Clustering result after clonal expansion.

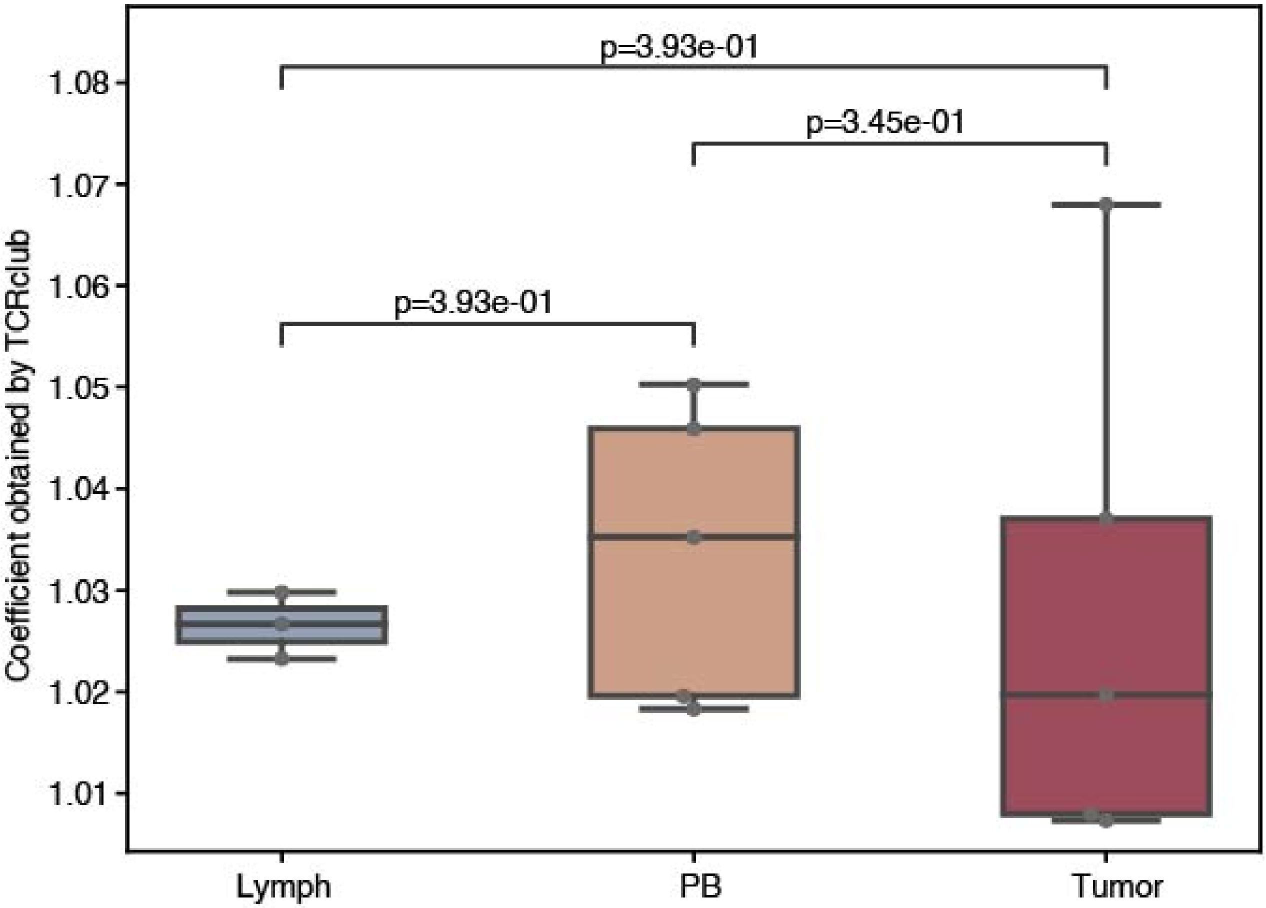

Appendix Figure S6. Comparison of the coefficients obtained by TCRclub across different tissues. The coefficients were averaged for each sample. P-value was derived from a one-sided Mann-Whitney's U-test.

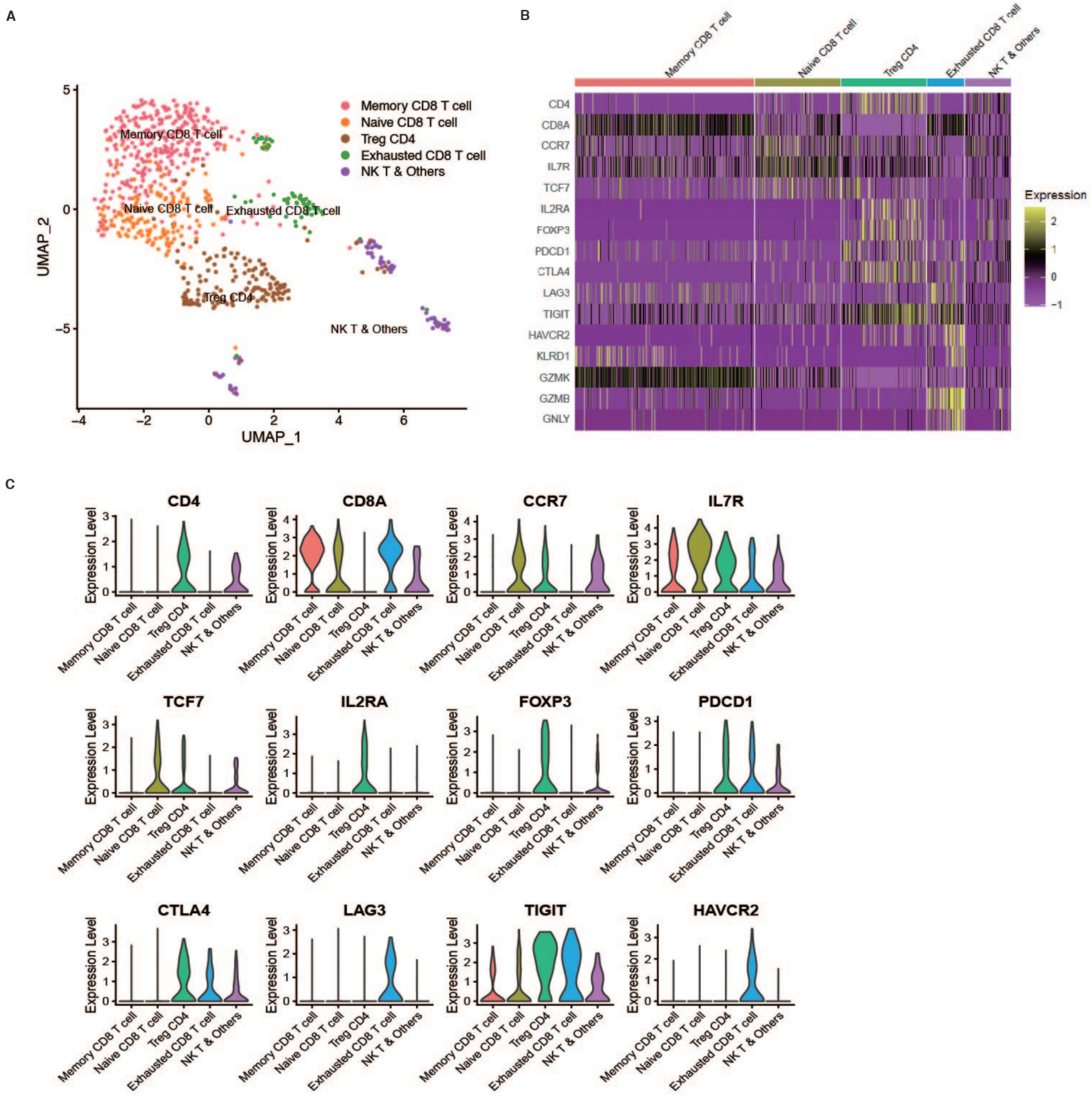

Appendix Figure S7. Clustering and subtype analyses of T cells in lymph node samples. We annotated the cells based on the annotation guidelines outlined previously by Shi et al. **a**, UMAP plot of subclustered T cells in lymph node samples, labeled in different colors. **b**, Heatmap of the marker genes for distinct immune cell subtypes.

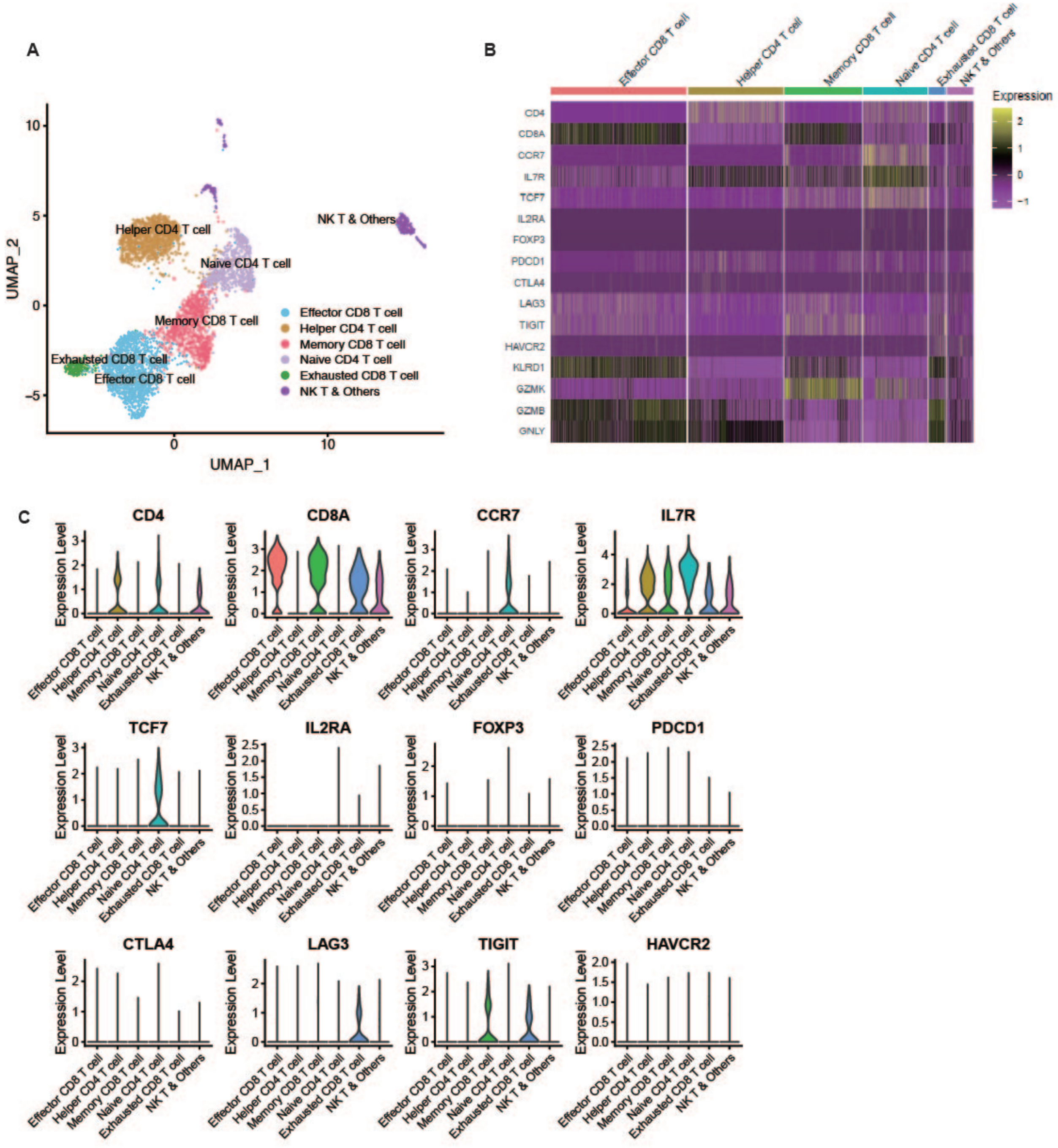

Appendix Figure S8. Clustering and subtype analyses of T cells in PB samples. We annotated the cells based on the annotation guidelines outlined previously by Shi et al. **A**, UMAP plot of subclustered T cells in PB samples, labeled in different colors. **B**, Heatmap of the marker genes for distinct immune cell subtypes.

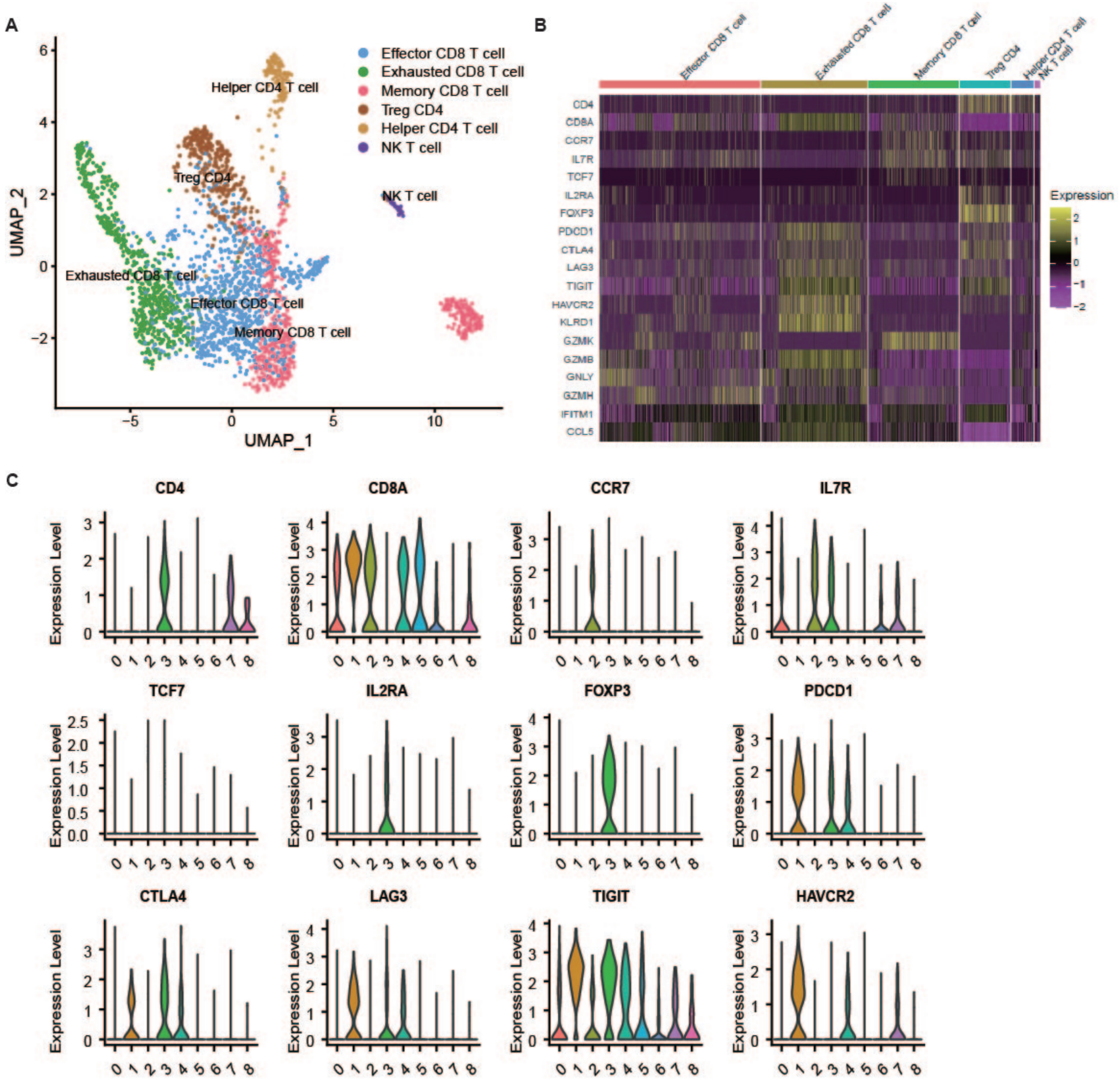

Appendix Figure S9. Clustering and subtype analyses of T cells in tumor primary locus samples. We annotated the cells based on the annotation guidelines outlined by Shi et al. **A**, UMAP plot of subclustered T cells in tumor primary locus samples, labeled in different colors. **B**, Heatmap of the marker genes for distinct immune cell subtypes.

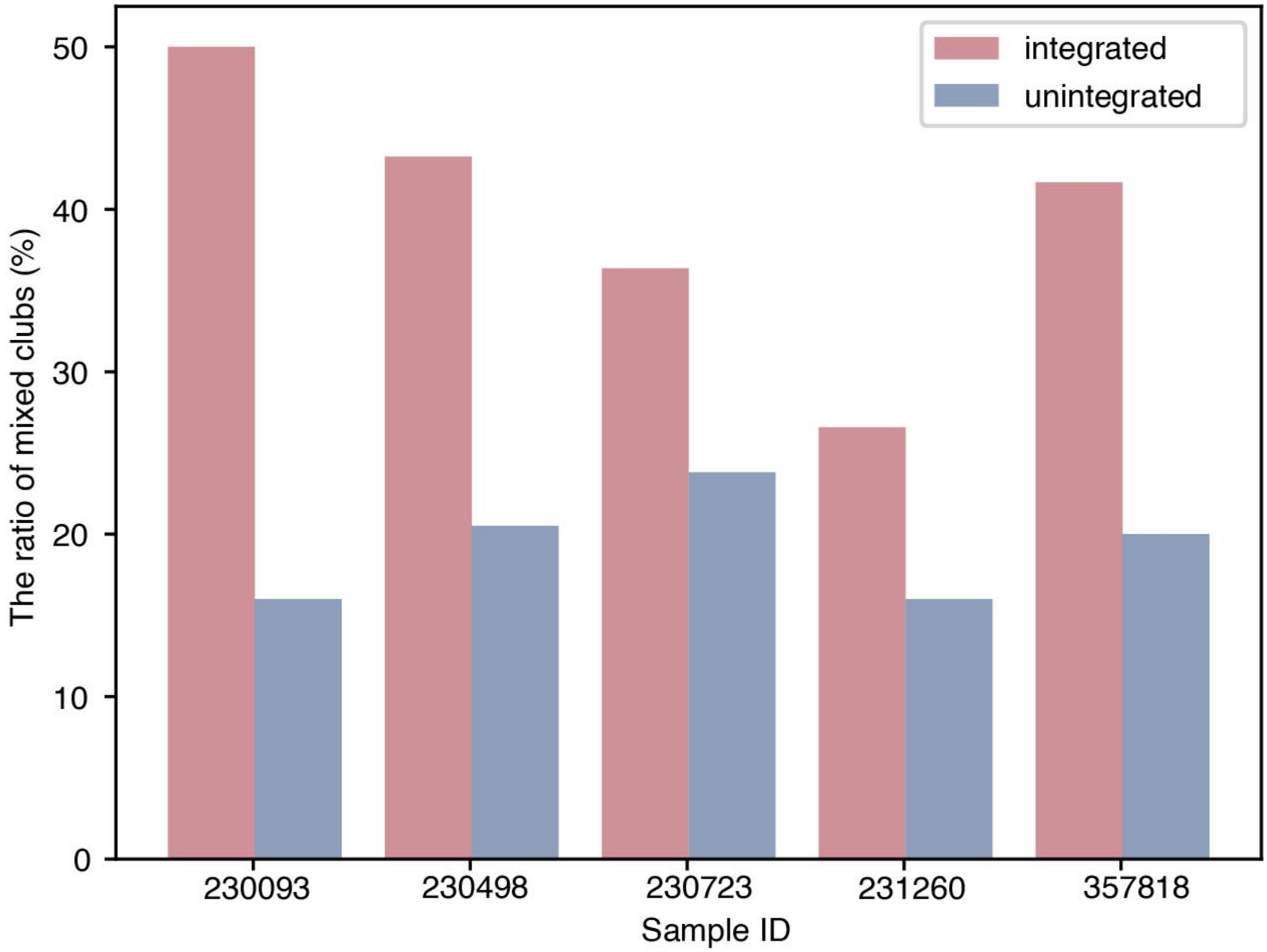

Appendix Figure S10. The comparison of the ratio of mixed clubs between integrated samples and unintegrated samples. Mixed clubs means the clubs containing T clones from different samples. The ratio of mixed clubs is calculated by the number of mixed clubs divided by the total number of clubs (clubs with only one T clone not included).

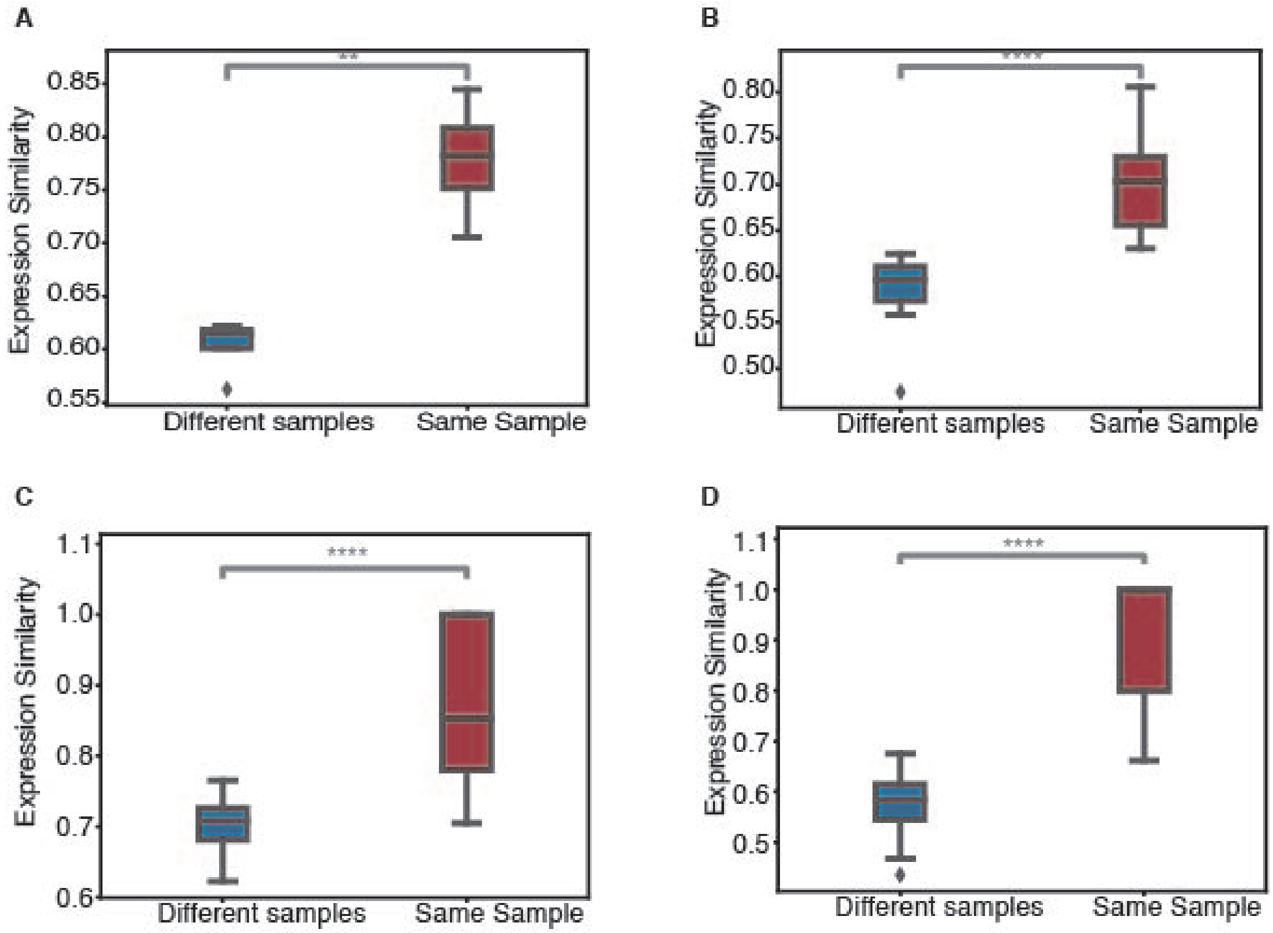

Appendix Figure S11. Expression similarity between T cells sharing identical CDR3β within the same sample and across different samples. Gene expressions from different samples were integrated, and the expression similarity of T cells sharing identical CD3β sequences was compared both within the same sample and across different samples. Four representative examples are presented. P-value was derived from a one-sided Mann-Whitney's U-test. $^{**}P < 10^{-2}$. $^{****}P < 10^{-4}$. **A**, Tumor and peripheral blood samples from cholangiocarcinoma patient 230093. **B**, Peripheral blood and lymph node samples from cholangiocarcinoma patient 230498. **C**, Pre-treatment and post-treatment samples from basal cell carcinoma patient 001. **D**, Pre-treatment and post-treatment samples from basal cell carcinoma patient 004.

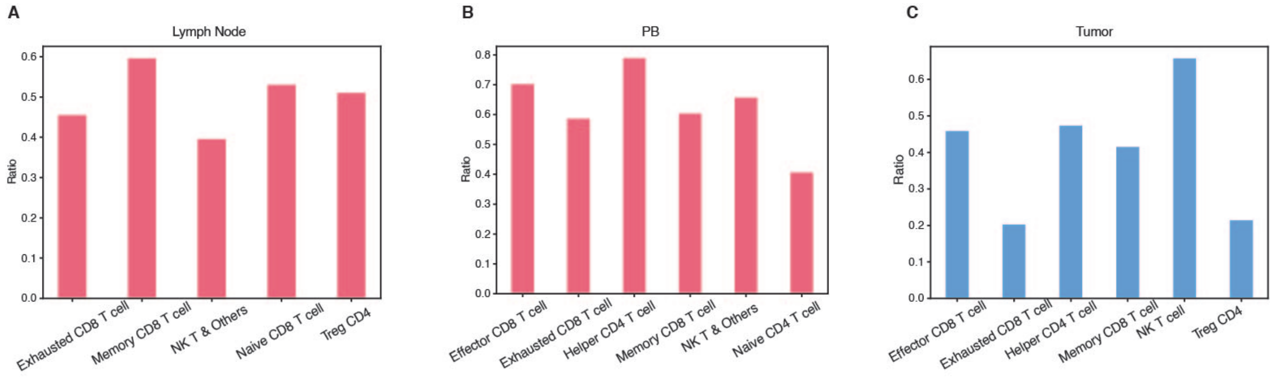

Appendix Figure S12. Ratio of tumor-related T cells (or PB-related T cells) to tumor-unrelated T cells (or tumor-unrelated T cells). **A**, Ratio of tumor-related T cells to tumor-unrelated T cells within lymph node samples. **B**, Ratio of tumor-related T cells to tumor-unrelated T cells within PB samples. **C**, Ratio of PB-related T cells to PB-unrelated T cells within tumor samples.

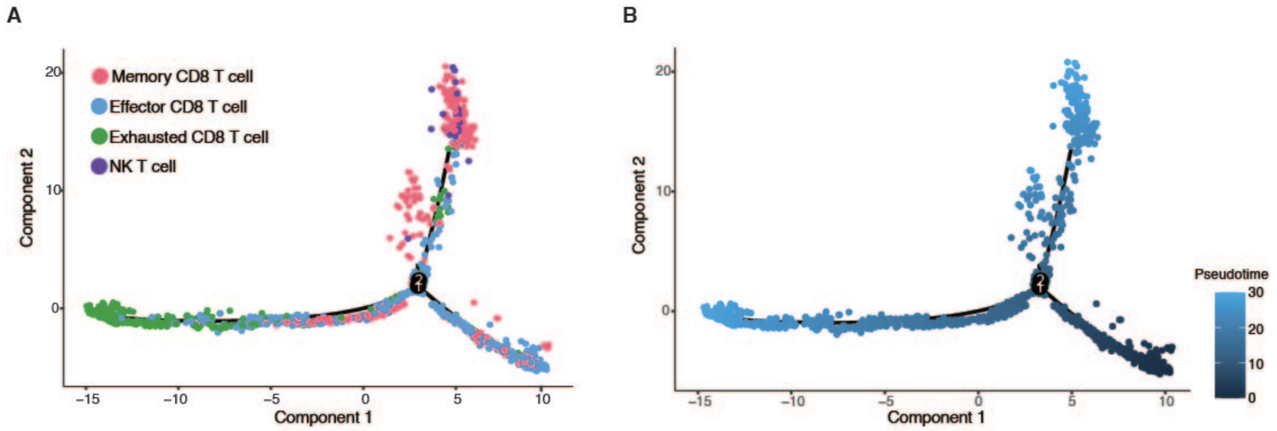

Appendix Figure S13. Trajectory and pseudotime analysis of CD8 T cells within tumor samples. A, Monocle 2 trajectory analysis illustrating CD8 T cells in tumor samples, annotated by cell subgroups. **B**, Monocle 2 pseudotime analysis of CD8 T cells.

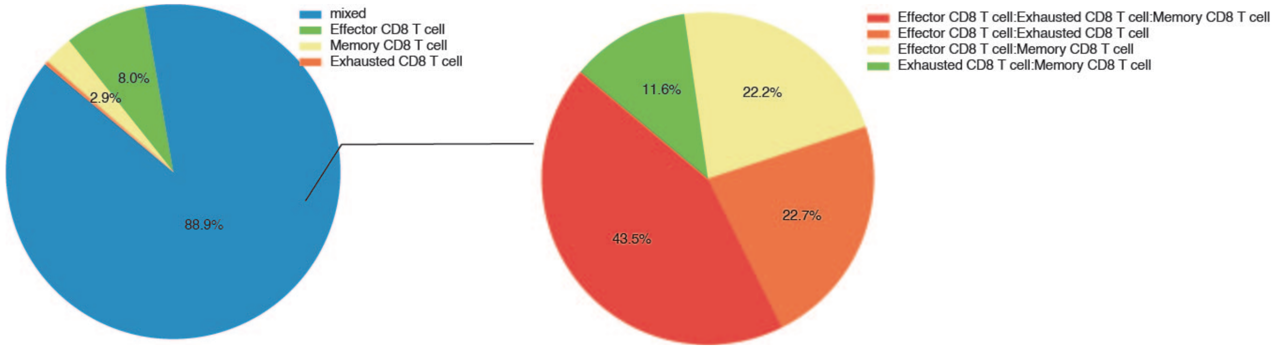

Appendix Figure S14. Analysis of the CD8 celltype distribution in the clubs for the T cells within tumor samples. Pie charts illustrates the distribution of CD8 celltypes in the clubs for the T cells within tumor samples. The left pie chart shows most of the clubs contain different CD8 celltypes. The right chart provides a detailed breakdown: among clubs with mixed CD8 celltypes, most of them contain the effector, memory and exhuasted CD8 celltypes at the same time.

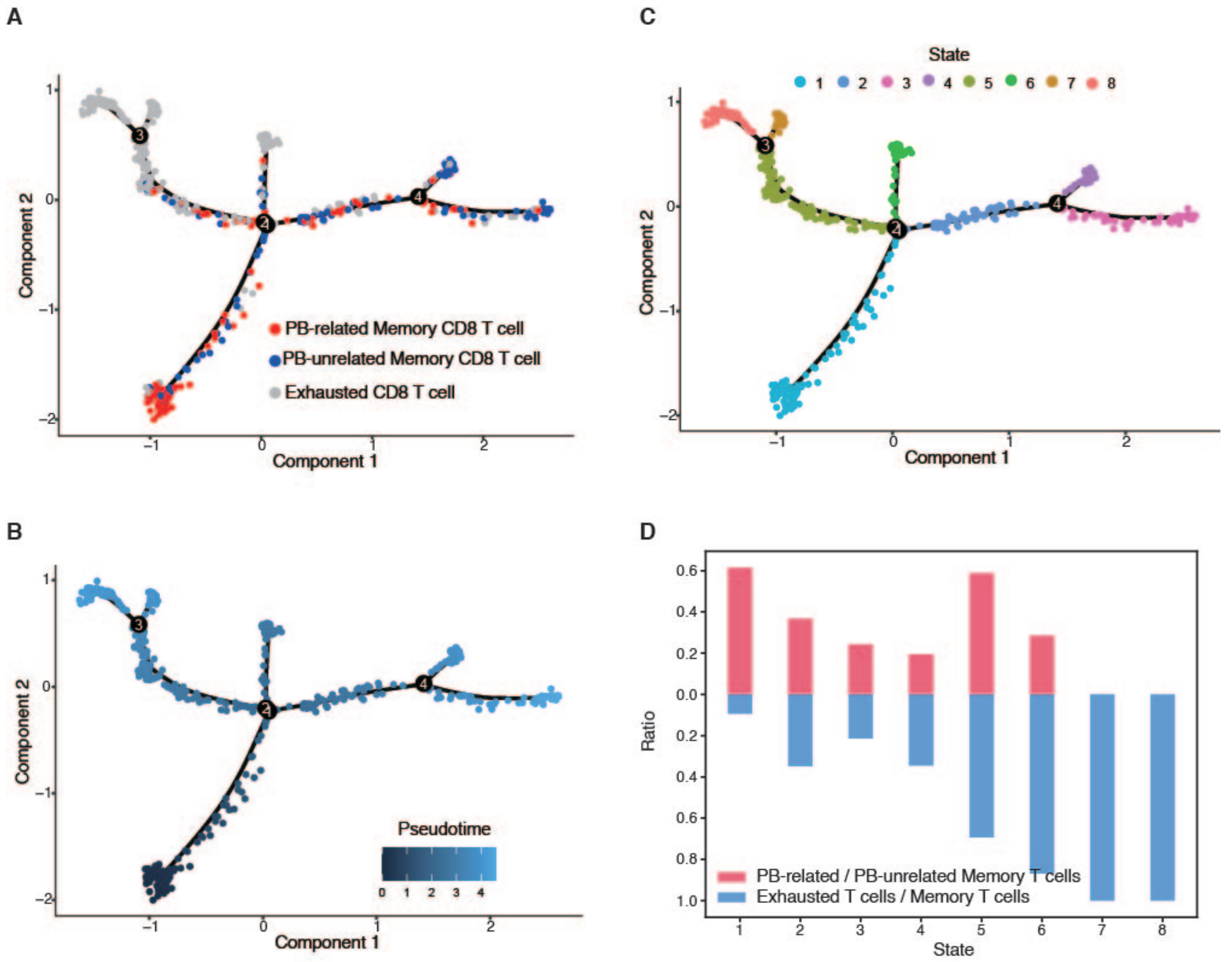

Appendix Figure S15. Trajectory and pseudotime analysis of memory CD8 T cells within tumor samples. A, Trajectory plot depicting the progression of memory CD8 T cells and exhausted CD8 T cells within tumor samples. **B**, Pseudotime trajectory plot of memory CD8 T cells and exhausted CD8 T cells within tumor samples. **C**, Trajectory plot demonstrating the states of memory CD8 T cells and exhausted CD8 T cells within tumor samples. **D**, Ratio of PB-related to PB-unrelated memory CD8 T cells, along with the ratio of memory CD8 T cells to exhausted T cells in each state depicted in **C**. Monocle 2 was employed for trajectory and pseudotime analysis.

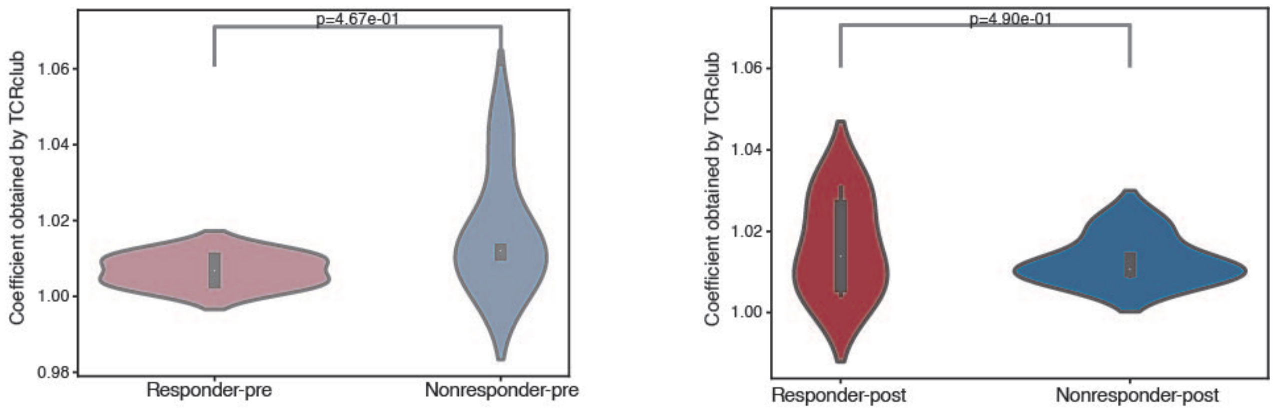

Appendix Figure S16. Comparison of the coefficients obtained by TCRclub across responders and nonresponders. The coefficients were averaged for each sample. P-value was derived from a one-sided Mann-Whitney's U-test.

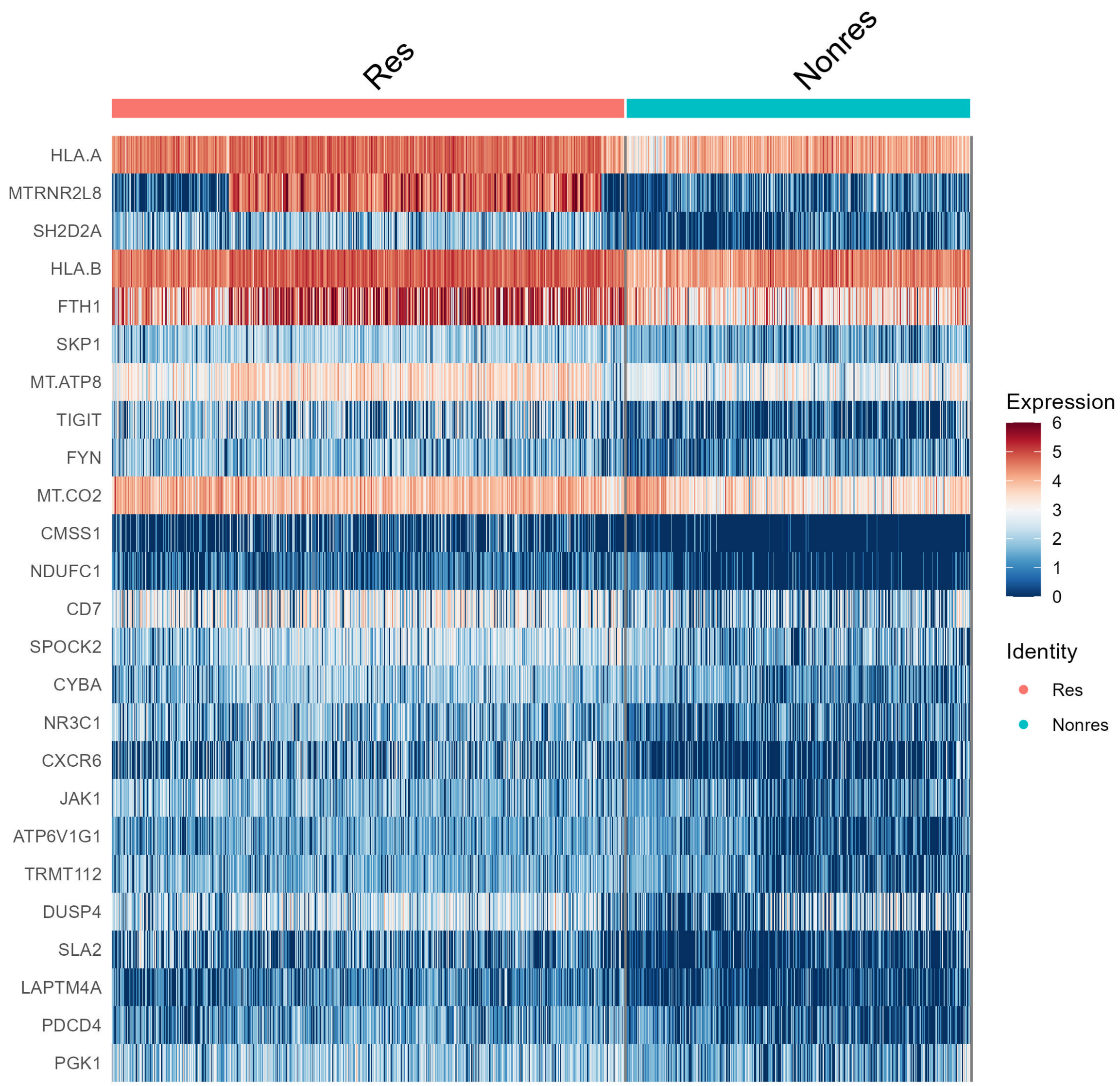

Appendix Figure S17. Heatmap for the differentially expressed genes discovered by analyzing the post-PD1 T cells that are related to the pre-PD1 T cells. The top 25 genes with the highest p-value in the responder group are shown.

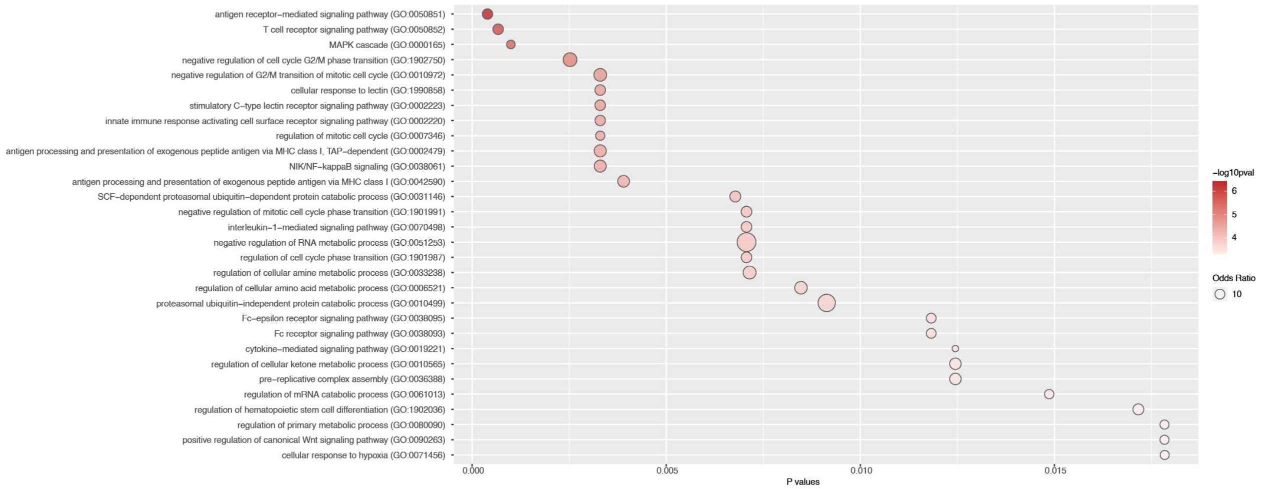

Appendix Figure S18. Top 30 Enriched Pathways in Responders. Pathway enrichment analysis was conducted, and the results were ranked based on their -log10pvalues. The figure displays the top 30 pathways identified in responders. P-values were generated from the two-sided Wilcoxon Rank Sum test.

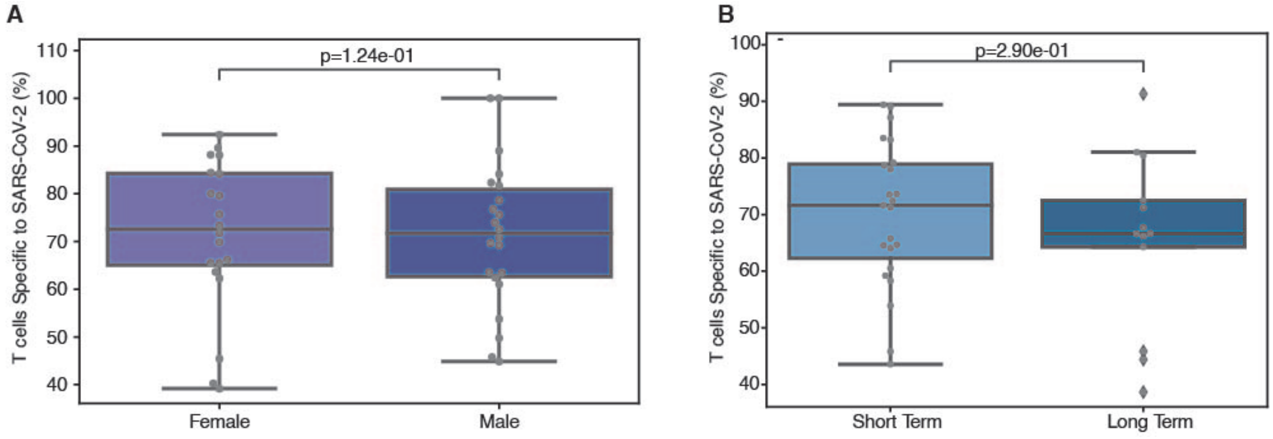

Appendix Figure S19. Distribution of SARS-CoV-2 antigen-specific T cells. A, Percentage of T cells specific to SARS-CoV-2 between male and female based on the clustering results from TCRclub. **B**, Percentage of T cells specific to SARS-CoV-2 between patients with short-term and long-term infection, based on the clustering results from TCRclub. Number of samples: Female ($n = 20$), Male ($n = 21$), Long-term ($n = 13$), Shot-term ($n = 23$), Long-term ($n = 13$). P-values were derived from a one-sided Mann-Whitney's U-test.

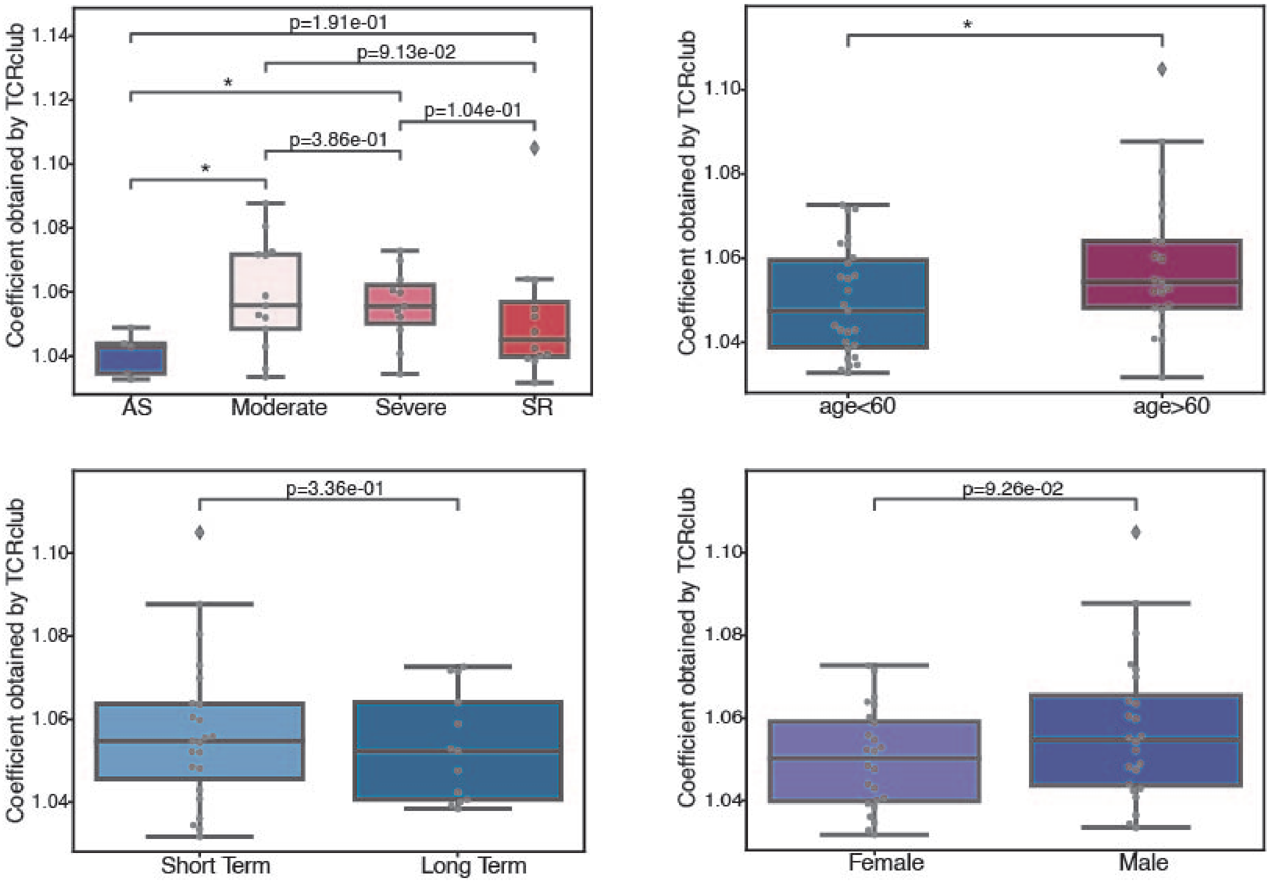

Appendix Figure S20. Comparison of the coefficients obtained by TCRclub across different symptoms. The coefficients were averaged for each sample. P-value was derived from a one-sided Mann-Whitney's U-test. $^*P < 0.05$.

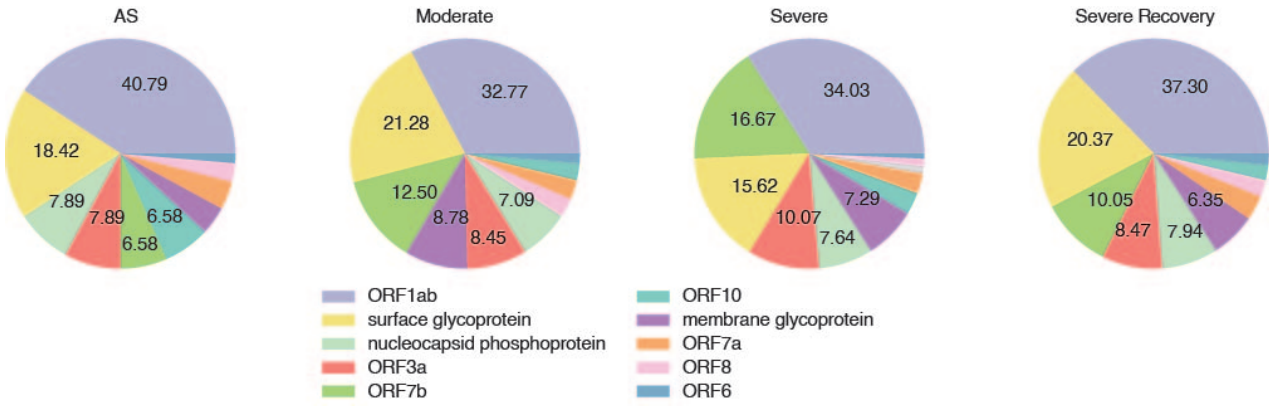

Appendix Figure S21. Distribution of different antigen-specific T-cell clubs for SARS-CoV-2. Pie chart of the distribution of antigen-specific T-cell clubs for SARS-CoV-2 across different severity levels.

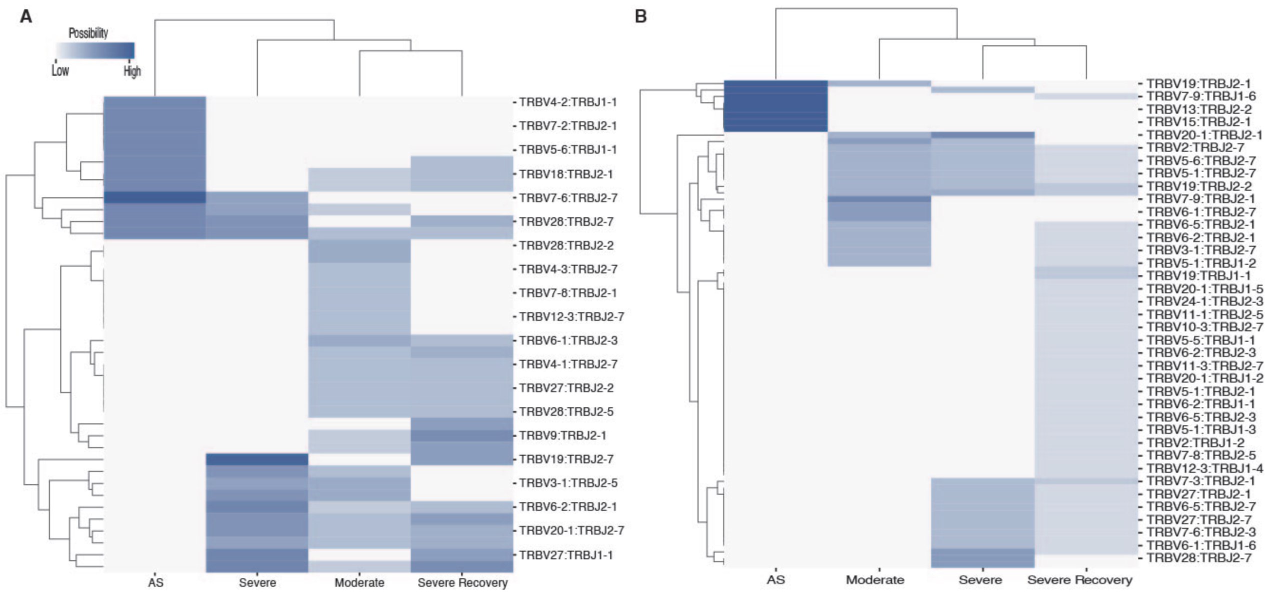

Appendix Figure S22. Most frequent V-J gene pairs. A-B, Most frequent V-J gene pairs for T cells specific to surface glycoprotein, the antigen that second most T-cell clubs specific to (**A**) and ORF7b, the antigen elicited fewer T-cell clubs' response (**B**).

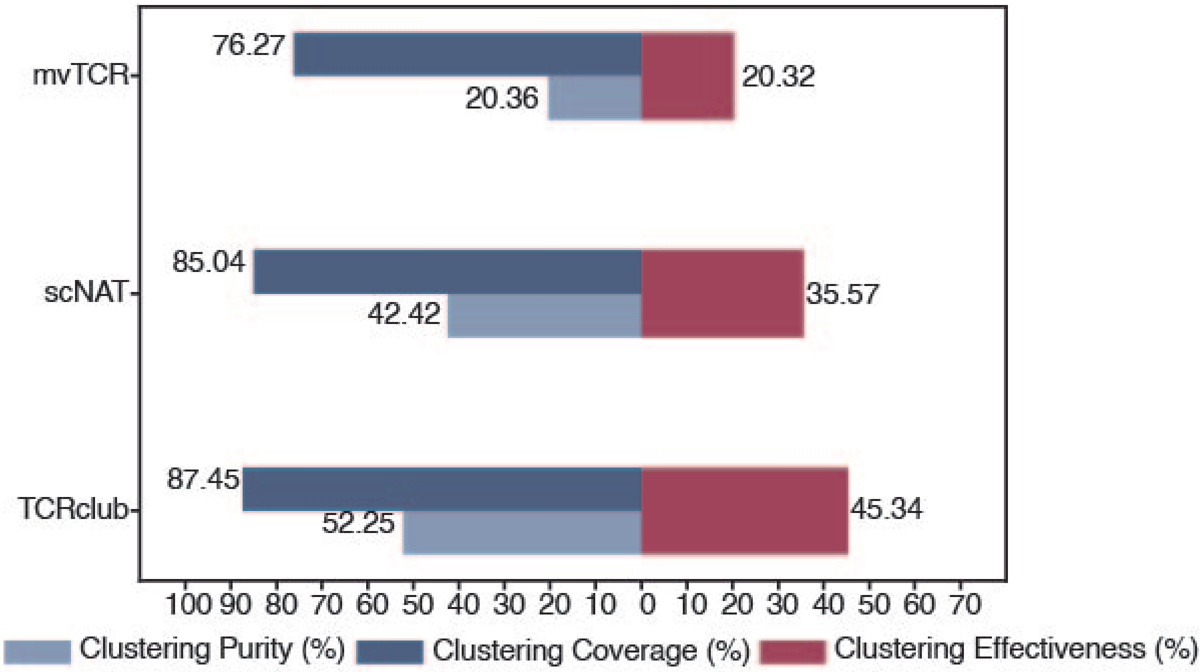

Appendix Figure S23. Comparison of integration performance with mvTCR and scNAT on the dataset with 400 pMHC categories. mvTCR and scNAT are tools designed to integrate scRNA-seq data with scTCR-seq data to generate embeddings for each T cell. The embeddings of T cells in each sample were obtained after at least 100 epochs of training. Since mvTCR and scNAT do not involve any clustering methods, after obtaining the embeddings for each T cell by their default setting, we applied our algorithm to identify T-cell clubs using pair-wise distances calculated from the embeddings (with $\zeta = 5 \times 10^{-4}$, consistent with TCRclub). Then, we compared the average clustering effectiveness, purity and coverage of the dataset.

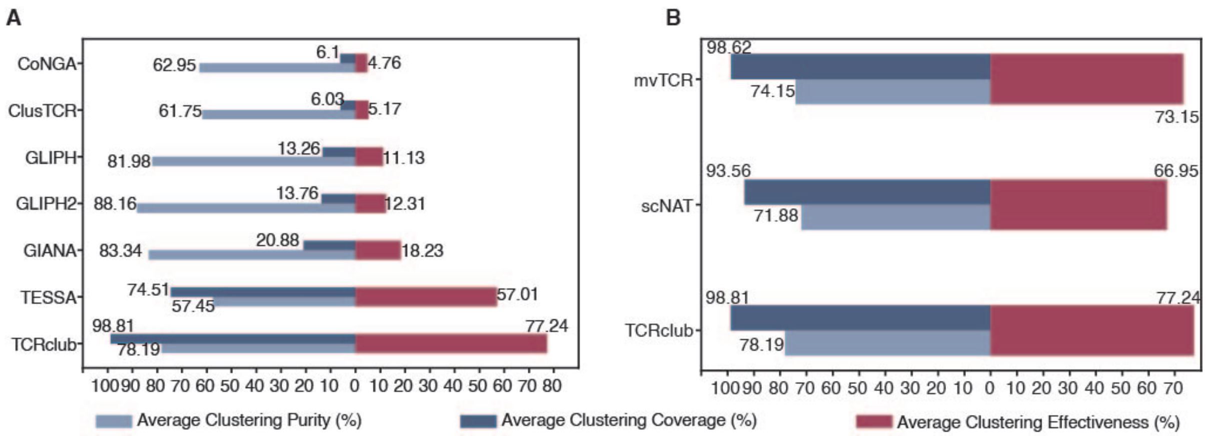

Appendix Figure S24. Comparison of the clustering performance for TCRclub and other models in healthy donor dataset. We applied different models to another dataset containing four samples and 44 pMHC categories from healthy individuals. If a method produced no T-cell cluster in a sample, then its purity and the coverage on the sample was regarded as zero. **A**, Clustering performance of TCRclub, TESSA, GIANA, GLIPH, ClusTCR and CoNGA by their default settings. **B**, Clustering performance of TCRclub, scNAT and mvTCR by their default settings. mvTCR and scNAT are tools designed to integrate scRNA-seq data with scTCR-seq data to generate embeddings for each T cell. The embeddings of T cells in each sample were obtained after at least 100 epochs of training. Because mvTCR and scNAT do not involve any clustering methods, after obtaining the embeddings for each T cell by their default setting, we applied our algorithm to identify T cell clubs using pair-wise distances calculated from the embeddings (with $\zeta = 5 \times 10^{-4}$, consistent with TCRclub). The average clustering effectiveness, purity, and clustering coverage of the dataset are presented.

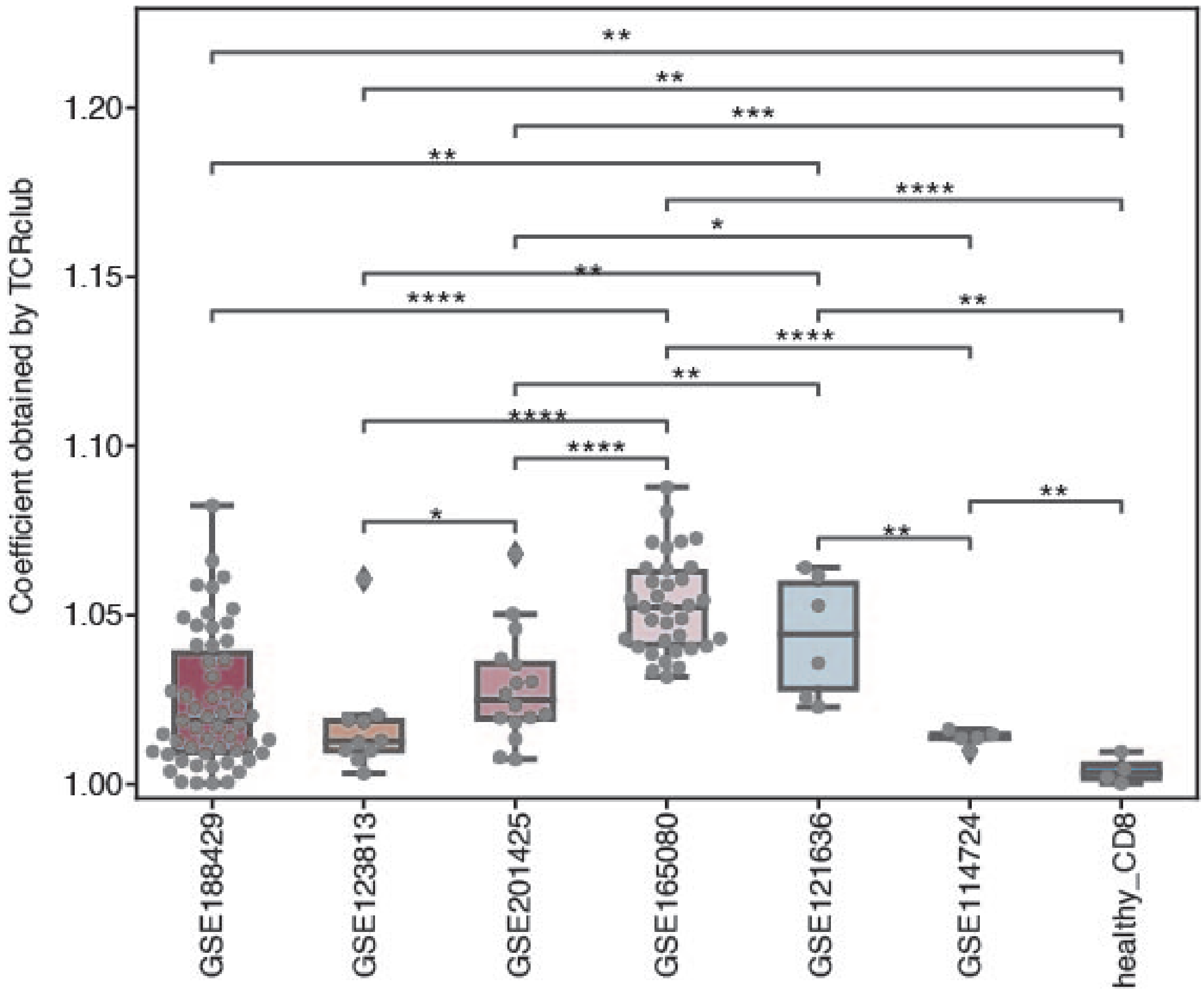

Appendix Figure S25. Comparison of the coefficients obtained by TCRclub across different datasets. The coefficients were averaged for each sample of the datasets (Appendix Table S1). The P-value was derived from a one-sided Mann-Whitney's U-test, and the P-values smaller than 0.05 are presented. $^{*}P < 5 \times 10^{-2}$. $^{**}P < 10^{-2}$. $^{***}P < 10^{-3}$. $^{****}P < 10^{-4}$.

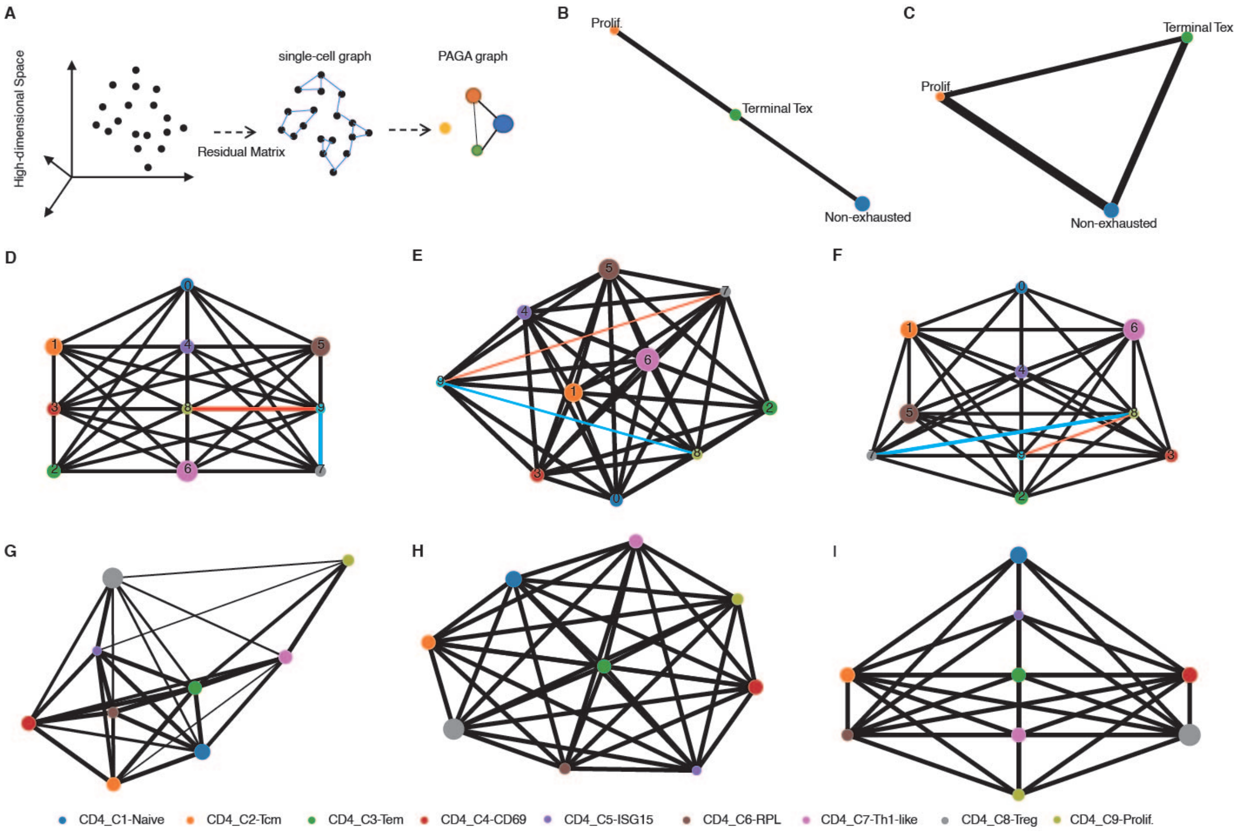

Appendix Figure S26. TCRclub extends the view of partition-based graph abstraction in drawing the topology of data. A study with this figure can be found in Appendix Supplementary Methods. **A**, Workflow demonstrating TCRclub's extension of the PAGA. **B-C**, PAGA graphs depicting CD8 T cell subtypes, with cells' kNN and distances defined by scRNA expression (**B**) and the residual-distance matrix in TCRclub (**C**). **D-F**, PAGA graph illustrating CD4 T cell subpopulations, with cells' kNN and distances defined by scRNA expression (**D**), scTCR embedding (**E**), and the residual-distance matrix in TCRclub (**F**). The thickness of the line represents the weight of the edges. **G-H**, PAGA graph displaying CD4 T cell subtypes, with cells' kNN and distances defined by scRNA expression (**G**), scTCR embedding (**H**), and the residual-distance matrix in TCRclub (**I**). The thickness of the line represents the weight of the edges.

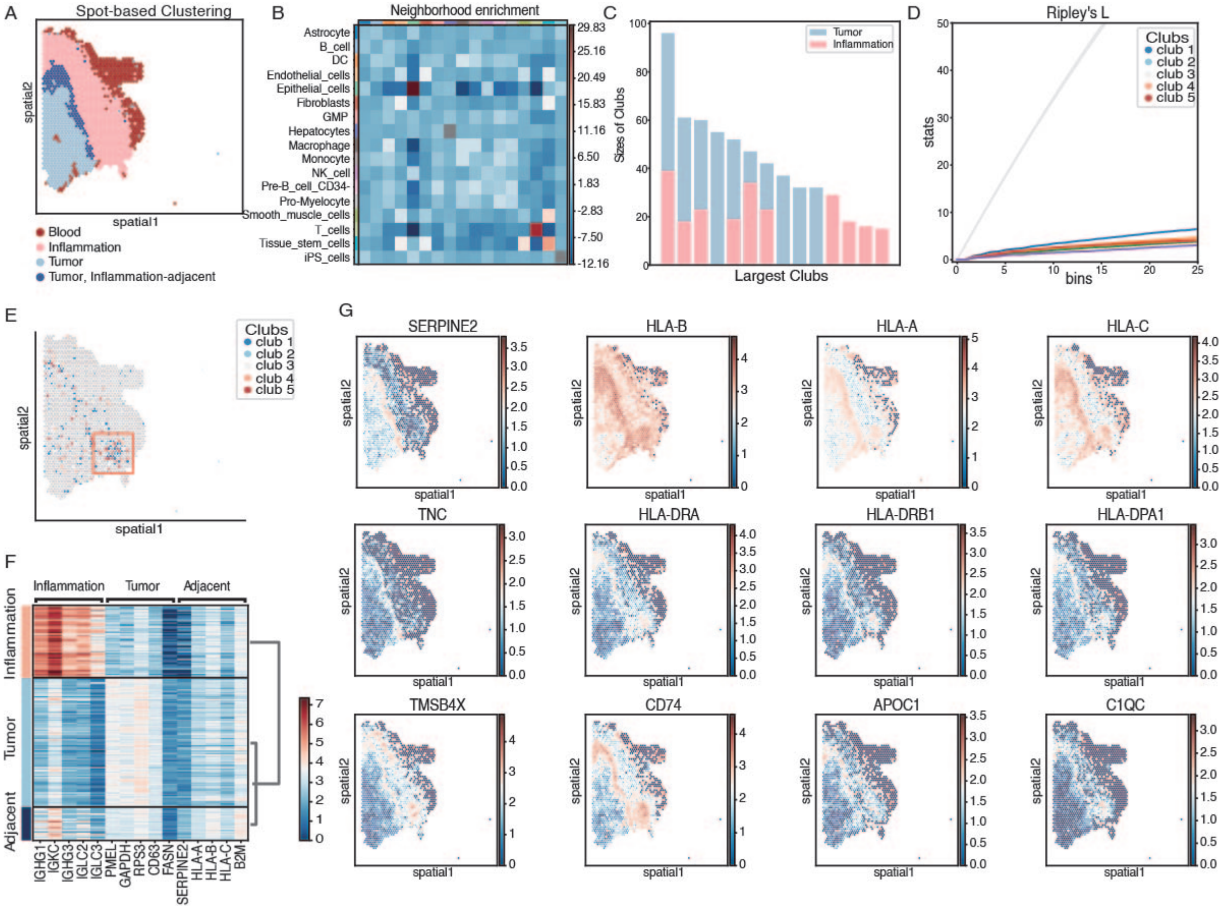

Appendix Figure S27. TCRclub reveals functionally-similar T cells are dispersed in tumor microenvironment of brain metastases. A study with this figure can be found in Appendix Supplementary Methods. **A**, Four primary areas. **B**, Heatmap for neighbourhood enrichment between different cell types. The neighbourhood enrichment test calculates the z-score to assess the interaction between two cell types. **C**, The largest clubs in tumor and inflammation area. Four clubs are consistently among the largest in the inflammatory and tumor areas. **D**, Ripley's L stats of five largest clubs in inflammation area. The dashed line indicates the value of Ripley'L for the expected random distribution. Deviations from the dashed line can indicate clustering (if above the line) or dispersion (if below the line) of the cells. **E**, Spatial distribution of five largest clubs in inflammation area. The concentrated area of the clubs near the tumor-inflammation adjacent area is circled by a red box. **F**, Differential expressed genes (DEGs) of inflammation, tumor, and tumor, inflammation-adjacent (Adjacent) areas next to the concentrated area in (**E**). Only the top five genes with the highest log2 fold change in each group are shown. **G**, Heatmap for the expressions of DEGs of the adjacent organisation in the area next to cluster.

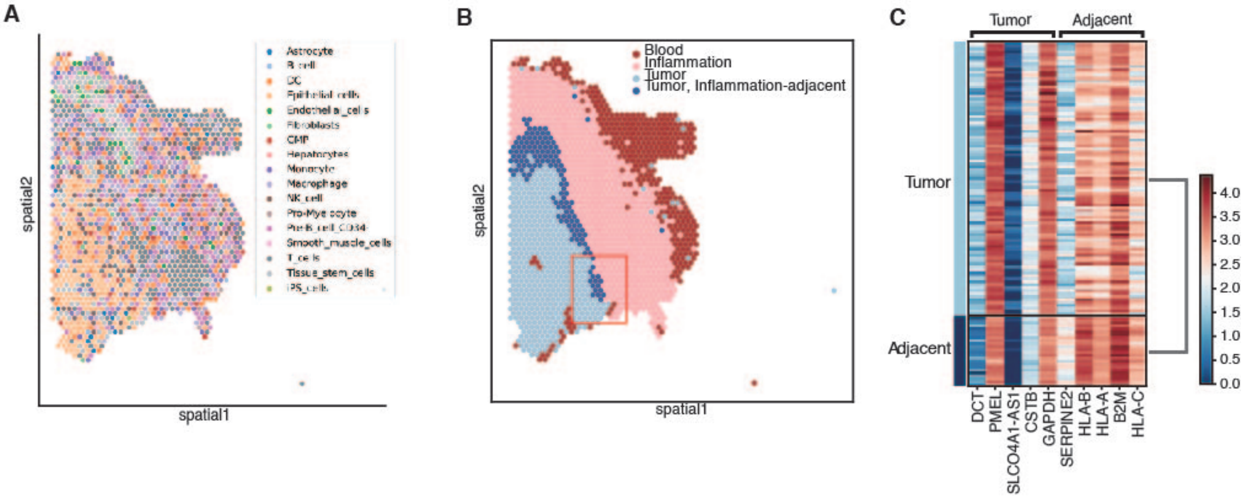

Appendix Figure S28. Spatial Distribution of Various Cell Types and Differentially Expressed Genes in the Adjacent Microenvironment. A study with this figure can be found in Appendix Supplementary Methods. **A**, Spatial distribution of different cell types within the tissue. **B**, Focused attention area near the concentration site, indicated by the red box. Differentially expressed genes within this attention area were analyzed. **C**, Comparison of differentially expressed genes between the tumor and adjacent tissue within the attention area. Only the top five genes with the highest log2 fold change in each group are shown.

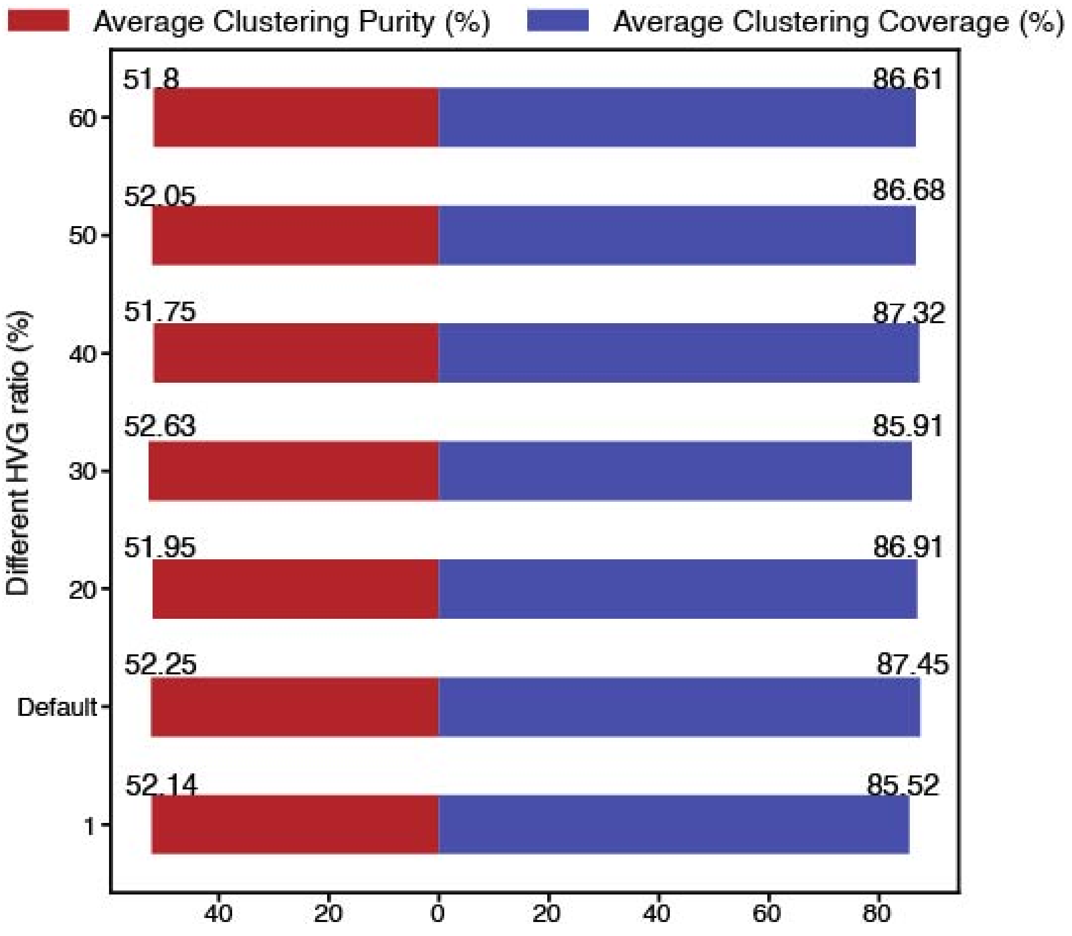

Appendix Figure S29. The clustering performance across different ratio of highly variable genes.

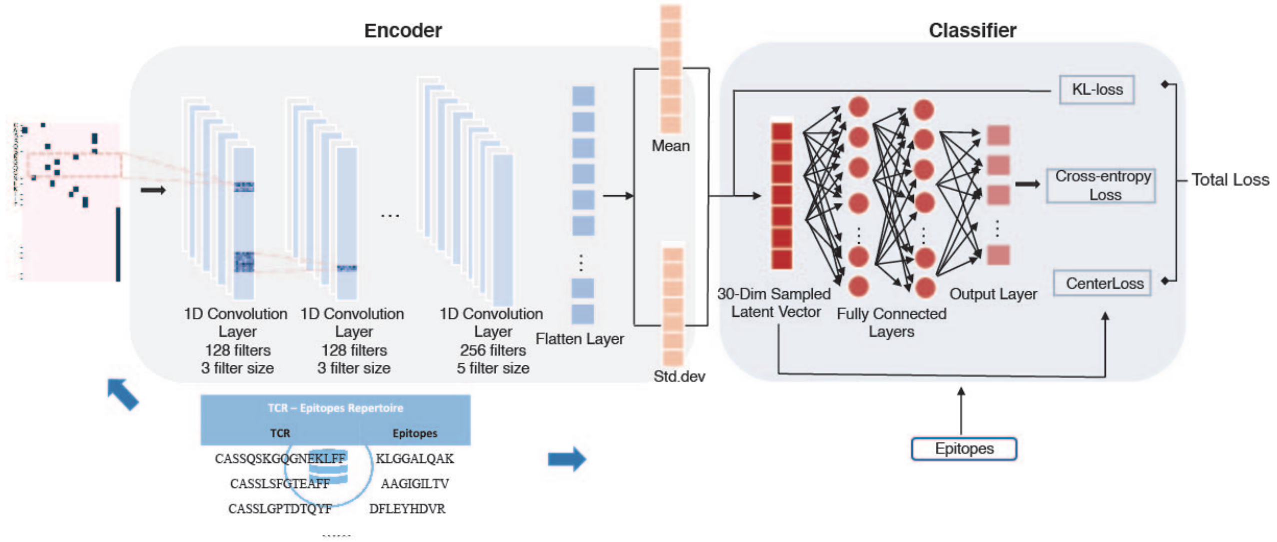

Appendix Figure S30. Schematic overview of the encoder-classifier for embedding TCR sequences.

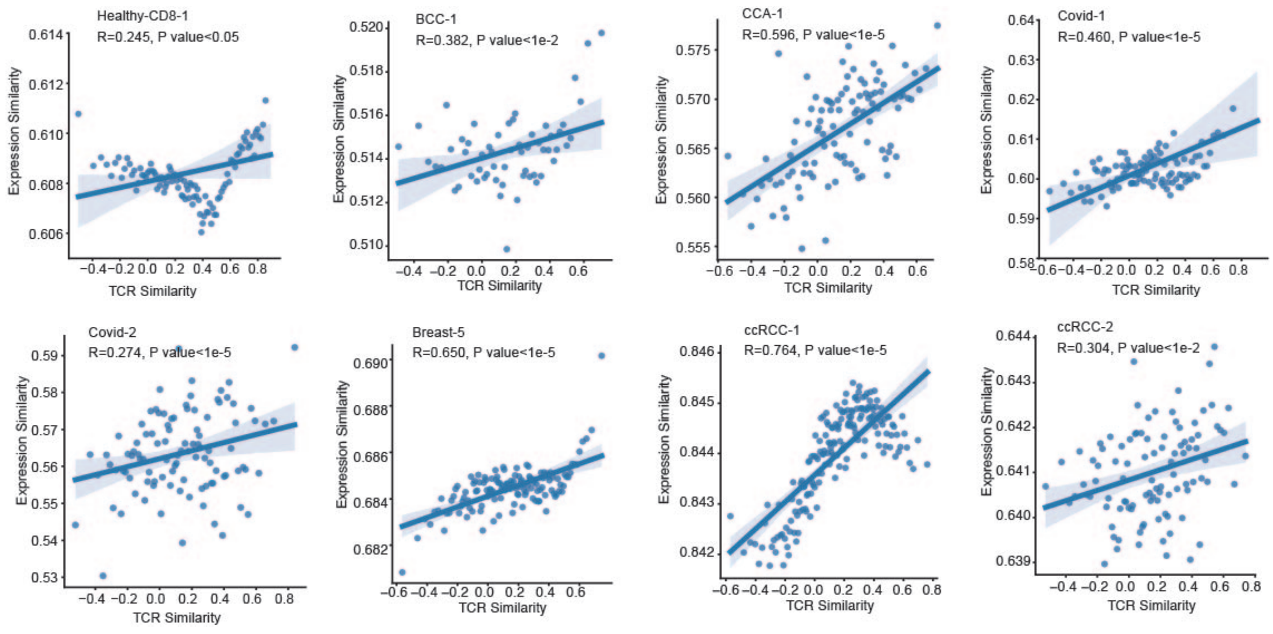

Appendix Figure S31. Scatterplots showing the relationships between the TCRs and RNA expression for several datasets. The gene expression distance between any two T cells was calculated using the dot product of the normalized gene expression vectors. Since the vectors were normalized, the dot product is equivalent to the Euclidean distance, meaning a larger dot product indicates greater similarity or a smaller distance. We calculated the gene expression distances of any two T-cell clones by averaging the gene expression distances within the T-cell clones. Similarly, the pairwise TCR embedding distance between any two of the T-cell clones was computed as the dot product of the normalized TCR embeddings. Several examples from various datasets are presented here. The P values indicate the significance of Pearson correlation coefficients. The shaded areas denote the 95% confidence intervals for linear regressions.

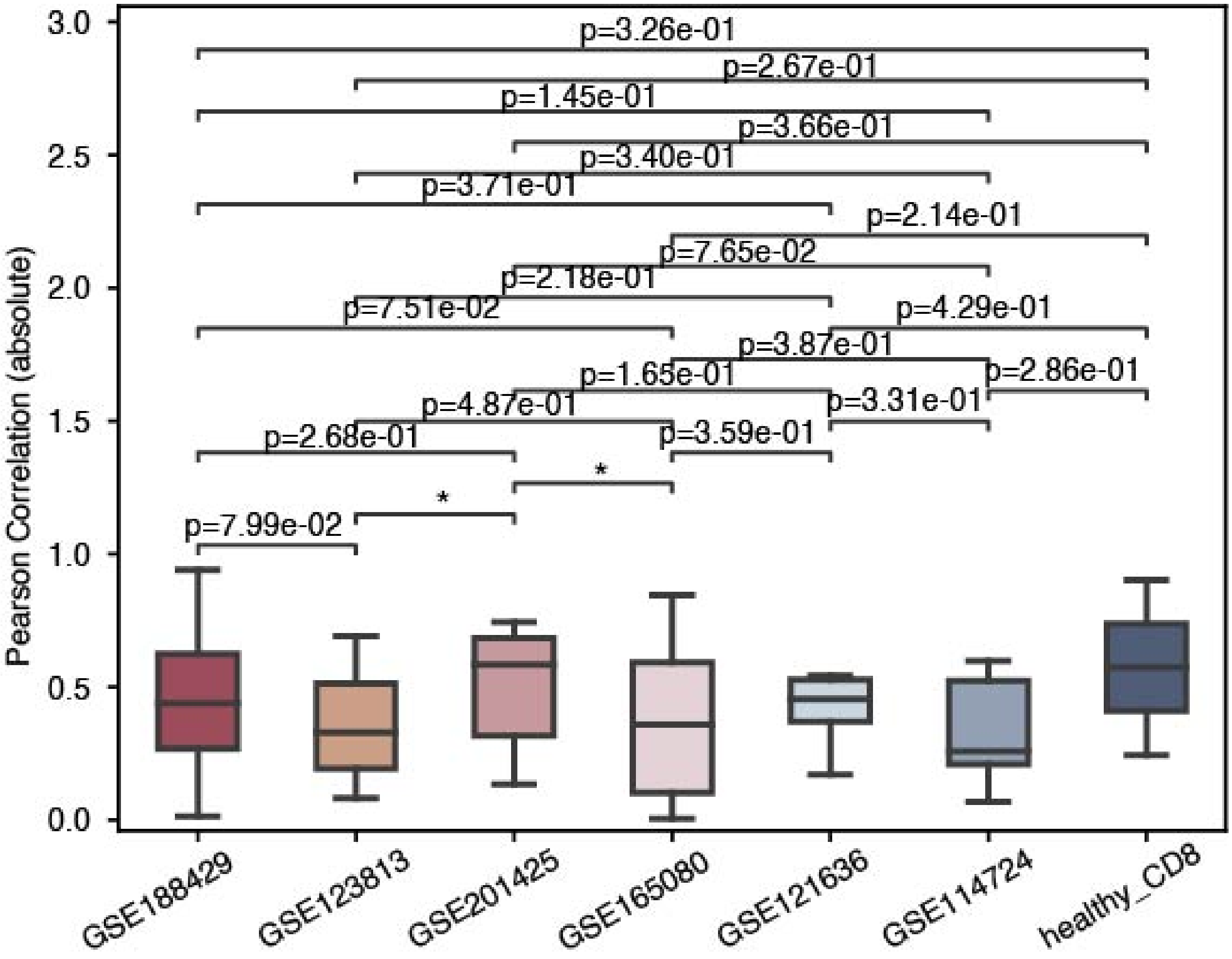

Appendix Figure S32. Pearson correlation between pairwise RNA distances and TCR embedding distances across datasets. Following gene expression preprocessing and TCR embedding generation, we calculated the Pearson correlation between pairwise RNA distances and pairwise TCR embedding distances for T-cell clones across multiple datasets (Appendix Table S1). The average value is 0.42 across all datasets. The P-value was derived from a one-sided Mann-Whitney's U-test. $^*P < 5 \times 10^{-2}$.

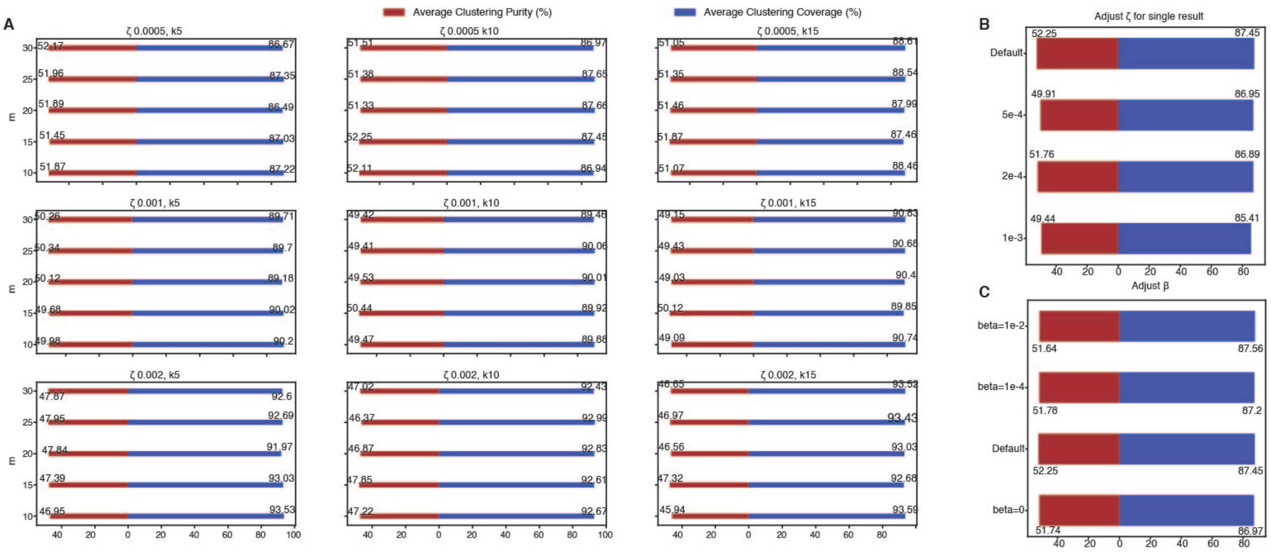

Appendix Figure S33. TCRclub Hyperparameter Settings. A, Trials for different settings of k, m, and consensus ζ. **B**, Trials for different settings of ζ for individual results. **C**, Trials for different settings of β. In each figure, parameters without specified values are set to their default settings.

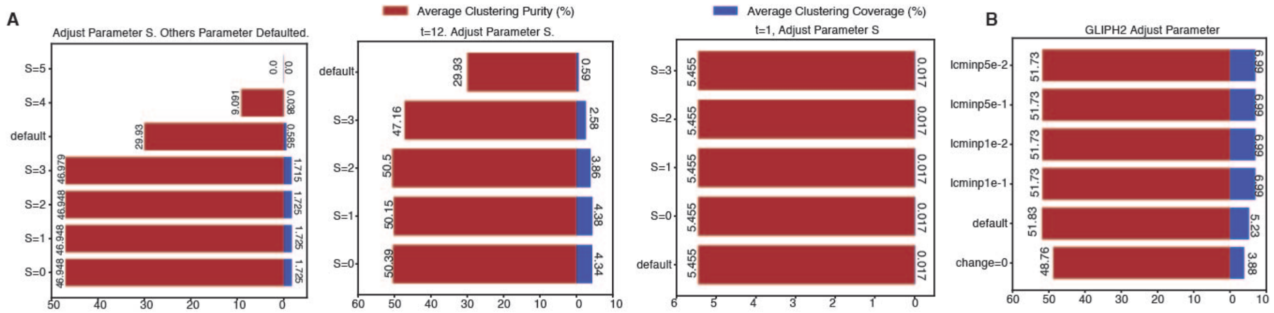

Appendix Figure S34. Adjusting parameters for other evaluated methods. We adjusted the parameters (if applicable) provided to update the clustering performance based on the documentation within their respective codes. Since we did not find a parameter interface provided by the documentation of TESSA's code, we only adjusted the parameters for GIANA and GLIPH2. **A**, Parameter adjustment for GIANA. Following the documentation of GIANA, we attempted to adjust parameters S and t to enhance its performance. Other unspecified parameters remained at their default settings. **B**, Parameter adjustment for GLIPH2. Based on the documentation of GLIPH, we attempted to adjust parameters *lcminp* and *change* to improve its performance. Other unspecified parameters retained their default settings.

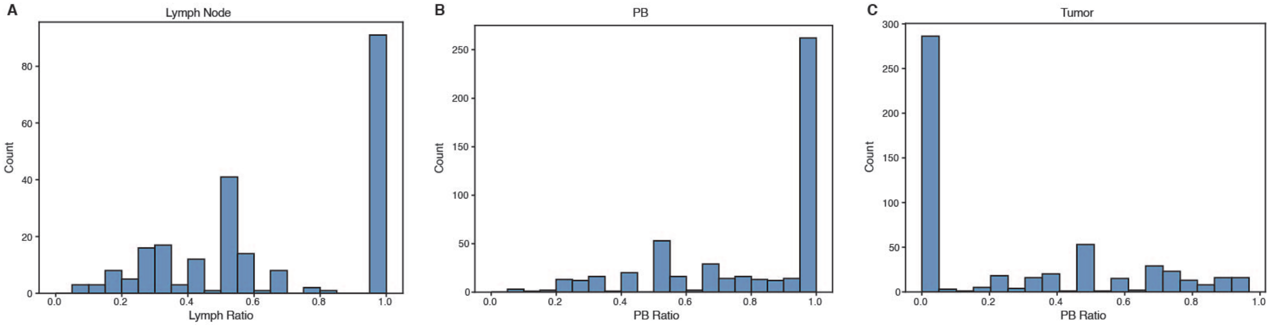

Appendix Figure S35. Distribution of clubs in integrated data. A, Distribution of the clubs containing lymph cells in lymph-tumor integrated data. The x-axis indicates the proportion (maximum value of 1) of lymph cells in a club, with the y-axis denoting the number of clubs falling within each range. **B**, Distribution of the clubs containing PB cells in PB-tumor integrated data. The x-axis denotes the proportion (maximum value of 1) of PB cells in a club, while the y-axis represents the number of clubs falling within each range. **C**, Distribution of the clubs containing tumor cells in PB-tumor integrated data. The x-axis represents the proportion (maximum value of 1) of PB cells in a club, while the y-axis indicates the number of clubs falling within each range. The bin-width is 0.05.

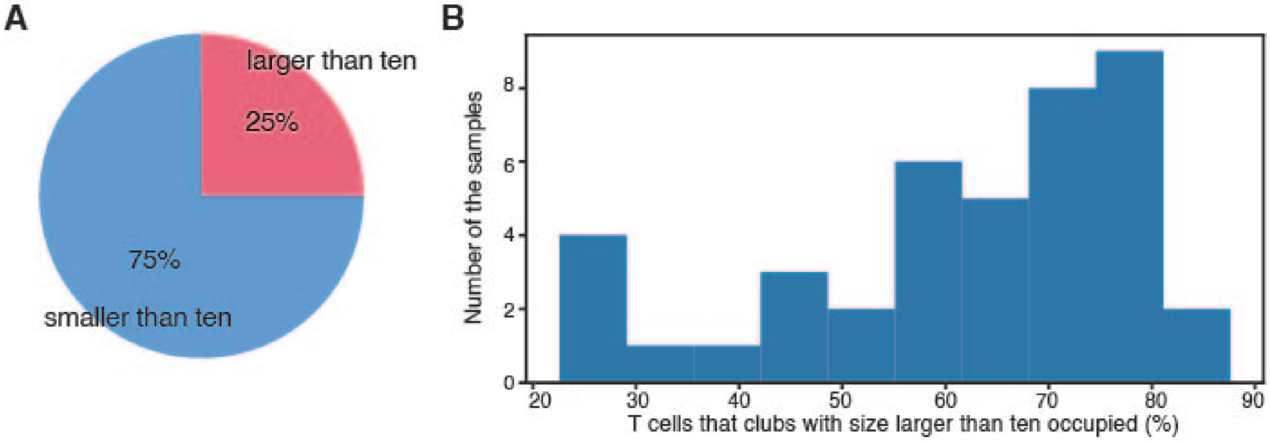

Appendix Figure S36. Distribution of the clubs with sizes larger than ten. A,Pie chart showing the percentage of clubs with sizes greater than ten. **B**, Histograms for the percentage of T cells occupied by the clubs with sizes larger than ten.

Supplementary Methods

Embedding CDR3β sequences

We trained a neural network model with an encoder-classsifier structure to generate discriminative embeddings, which are vectors of 32 dimensions, for the CDR3β amino acid sequences. The principal and structure design of the model has been described previously (Luo et al., 2023). In essence, the network consists of a probabilistic encoder based on the Variation Autoencoder (Kingma and Welling, 2013) (VAE) and a classifier for the targeted epitope classes of TCRs, as shown in Appendix Figure S27. We selected TCR sequences with single-specificity (i.e., removing TCR sequences known to match multiple epitopes) from the IEDB databases (Vita et al., 2019), resulting in 107,042 unique TCR sequences against 627 epitopes in total. The training dataset and the principal and structure design of the model have been described previously (Luo et al., 2023). We adapted the original model to fit our study.

Let f denote the number of dimensions in embedding. To prepare the CDR3β sequences as input of our neural network, we convert each sequence into a one-hot encoded matrix, also known as one-hot embedding. The maximum length of the input sequence is set as l, and any sequence shorter than l is padded to match this length. Each one-hot encoded matrix has a size of $l \times 21$, where the first 20 columns represent the 20 amino acids, respectively, and the last column indicates whether a position is padded. Assuming there are n CDR3β sequences in the mini-batch, we can represent the one-hot encoded matrix of CDR3β sequence i as $x_i \in \mathbb{R}^{l\times 21}$, where $i = 1, 2, \ldots n$.

The probabilistic encoder then encodes x_i into two variables: the mean $\mu_i \in \mathbb{R}^f$, and the standard deviation $\sigma_i \in \mathbb{R}^f$. The two variables are used to parametrize a normal distribution, and together they generate the latent vector $v_i \in \mathbb{R}^f$ for sequence i. v_i serves as the TCR embedding of sequence i in our study. The representation of v_i can be expressed as follows:

$$v_i = \mu_i + \sigma_i \odot \varepsilon, \quad \varepsilon \sim N(0, I). \tag{1}$$

To ensure that the generated latent vectors v are representative of the input sequences x, the encoder then optimizes the similarity between two distributions $p(v|x)$ and $p(x|v)$ using the Kullback-Leibler divergence L_{KL}, which is defined as follows:

$$L_{KL} = \sum_{i=1}^{n}\sum_{d=1}^{f}\frac{1}{2}[\mu_{id}^2 + \sigma_{id}^2 - (1 + log\sigma_{id}^2)]. \tag{2}$$

Next, we employ a classifier to achieve epitope classification, which makes the latent vectors of the TCRs with the same epitope-specificity closer. For a given input sequence x_i, let $y_i \in \mathbb{R}^E$ denote the one-hot vector of the ground truth, where E is the number of epitope classes in the TCR-Epitopes repertoire. Let $\hat{y}_i \in \mathbb{R}^E$ denote the predicted vector processed by the softmax function. To measure the dissimilarity between the predicted and ground truth vectors, we use the cross-entropy loss function $L_{cross-entropy}$, defined as

$$L_{cross-entropy} = -\sum_{i=1}^{n}\sum_{j=1}^{E} y_{ij} log(\hat{y}_{ij}). \tag{3}$$

To enhance the encoder-classifier's performance, we further incorporate the center Loss (Wen et al., 2016) to reduce intra-class variability among latent vectors. Let $C_{y_i} \in \mathbb{R}^f$ represent the center of the latent vectors associated with the epitope class y_i in a mini-batch dataset. The center loss function is defined as follows:

$$L_c = \frac{1}{2}\sum_{i=1}^{n} \|v_i - C_{y_i}\|^2. \tag{4}$$

Finally, the three loss functions, L_{KL}, $L_{cross-entropy}$, and L_c, comprise our encoder-classifier's objective function L_{total}. We used two hyperparameters, α and β, to adjust the relative weighting of L_{KL} and L_c in L_{total}, respectively. The complete expression for L_{total} is given as

$$
\begin{aligned}
L_{total} &= \frac{1}{n}(L_{cross-entropy} + \alpha L_{KL} + \beta L_c) \\
&= -\frac{1}{n}\sum_{i=1}^{n}\left\{\sum_{j=1}^{E} y_{ij} log(\hat{y}_{ij})\right. \\
&\quad + \sum_{d=1}^{f}\frac{\alpha}{2}\left[\mu_{id}^2 + \sigma_{id}^2 - (1 + log\sigma_{id}^2)\right] \\
&\quad \left. + \frac{\beta}{2}\|v_i - C_{y_i}\|^2\right\}.
\end{aligned} \tag{5}
$$

Initialization of TCRclub parameters

Fixed Initialization of Parameter C

The elements in the diagonals of W and A are uniformly initialized as 1. Users can choose the fixed initialization for parameter C if the consensus result is unnecessary. In the method of fixed initialization, the calculation of the possibility matrix P is removed. Instead, we directly selected k nearest neighbours for each cell. Let ϕ_i denote the k selected neighbours of cell i. The elements of the row vector C_i are initialized as follows:

$$
C_{ij} = \mathbb{I}(j \in \phi_i) \quad j = 1, 2, \cdots, n. \tag{6}
$$

Random Initialization of Parameter C

This initialization of C is more random. Assume cell j is one of the cell i's k nearest neighbours according to the initial-residual matrix $\hat{\Delta}$, and $C'_{ij} = 1$; It is noted that cell i could not be the nearest neighbours of cell j and the case ($C'_{ij} = 0$ while $C'_{ji} = 1$) can occur. Therefore, C' is a directed adjacency matrix of the cell-cell graph. To add more undirected connections, we create the symmetric matrix $\hat{C}$ as follows:

$$
\hat{C}_{ij} = \mathbb{I}(C'_{ij} + C'_{ji} \geq 1) \quad i, j = 1, 2, \cdots, n. \tag{7}
$$

We define cell q as a newly added cell to cell p if $C'_{pq} = 0$, $C'_{qp} = 1$, and $\hat{C}_{pq} = 1$. It should be noted that such newly added cells could lead to bad initialization performance if $\hat{S}_{pq}$ is much larger than the similarity-distance of cell p's k nearest neighbours.

To filter out such outliers in the newly added cells, we set a parameter k_*, $k_* \geq k$. we set the default k_* as k. For a cell i, $i = 1, 2, \cdots, n$, let φ_i denote the set of k_* nearest neighbours for cell i, and φ_i represents the set of corresponding initial-residual elements $\hat{\Delta}_{ih}$, where $h \in \varphi_i$. We assume a probability matrix P for $\hat{C}$, where $P \in \mathbb{R}^{n \times n}$. Specifically, P_{ij} can be calculated as follows:

$$
\begin{aligned}
P'_{ij} &= \begin{cases} 1/\hat{C}_{ij}\hat{\Delta}_{ij}, & if\ \hat{\Delta}_{ij} \leq \hat{\Delta}_{i_{Q_3}} + 1.5\hat{\Delta}_{i_{IQR}}\ and\ \hat{C}_{ij}\hat{\Delta}_{ij} > 0 \\ 0, & if\ \hat{S}_{ij} \geq \hat{\Delta}_{i_{Q_3}} + 1.5\hat{\Delta}_{i_{IQR}}\ or\ \hat{C}_{ij}\hat{\Delta}_{ij} = 0, \end{cases} \quad i, j = 1, 2, \cdots, n \\
P_{ij} &= P'_{ij}/\textstyle\sum_{j=1}^{n} P'_{ij}, \quad i, j = 1, 2, \cdots, n,
\end{aligned} \tag{8}
$$

where $\hat{\Delta}_{i_{Q_3}}$ and $\hat{\Delta}_{i_{IQR}}$ are the third quartile and the interquartile range of $\hat{\Delta}_i$, respectively. Then, for a cell i, $i = 1, 2, \cdots, n$, we sample k_* cells according to the row i of probability matrix P. The larger P_{ij} is, the more likely the cell j is selected. We sample k neighbours according to the matrix P for each cell. Let ϕ_i denote the k sampled neighbours of cell i. The elements of the row vector C_i are initialized as follows:

$$
C_{ij} = \mathbb{I}(j \in \phi_i) \quad j = 1, 2, \cdots, n. \tag{9}
$$

Default Initialization of Parameter C

We adopted random initialization as the default for parameter C due to various factors. Different initial points may converge to disparate local minima, and fixed initialization can predispose to suboptimal clustering by failing to comprehensively explore the parameter space, potentially missing the global optimum. By integrating random initialization, we introduce diversity in starting points, thereby improving the likelihood of identifying the global minimum (Swirszcz et al., 2016; Shahriari et al., 2022; Lee and Stöger, 2023). Subsequently, to enhance the stability and robustness of our clustering algorithm, TCRclub employs a bagging-like approach by repeating the process multiple times to obtain a consensus result. This iterative process reduces algorithm variance stemming from random initialization, mitigates overfitting, and enhances generalization capabilities (Breiman, 1996; Dudoit and Fridlyand, 2003).

Solutions of TCRclub parameters

We solved for W, A and C in turn in every iteration. Let L denote the objective function and W_{mm} represent the m element in the diagonal of W. The analytical solution of W_{mm} can be obtained from,

$$\begin{gathered}\frac{\partial L}{\partial W_{mm}} = 0, \\ \sum_{i,j=1}^{n} C_{ij} A_{ij}^2 (TWT^t)_{ij} \tau_{im} \tau_{jm} + \beta \tau_{mm} = \sum_{i,j=1}^{n} C_{ij} A_{ij} R_{ij} \tau_{im} \tau_{jm}.\end{gathered} \tag{10}$$

The analytical solution of matrix W can be expressed by

$$\begin{bmatrix} \sum_{i,j=1}^{n} C_{ij} A_{ii}^2 \tau_{i1}^2 \tau_{j1}^2 + \beta, & \sum_{i,j=1}^{n} C_{ij} A_{ii}^2 \tau_{i1} \tau_{j1} \tau_{i2} \tau_{j2}, & \cdots, & \sum_{i,j=1}^{n} C_{ij} A_{ii}^2 \tau_{i1} \tau_{j1} \tau_{if} \tau_{jf} \\ \sum_{i,j=1}^{n} C_{ij} A_{ii}^2 \tau_{i1} \tau_{j1} \tau_{i2} \tau_{j2}, & \sum_{i,j=1}^{n} C_{ij} A_{ii}^2 \tau_{i2}^2 \tau_{j2}^2 + \beta, & \cdots, & \sum_{i,j=1}^{n} C_{ij} A_{ii}^2 \tau_{i2} \tau_{j2} \tau_{if} \tau_{jf} \\ \vdots & \vdots & \ddots & \vdots \\ \sum_{i,j=1}^{n} C_{ij} A_{i1}^2 \tau_{i1} \tau_{j1} \tau_{if} \tau_{jf}, & \sum_{i,j=1}^{n} C_{ij} A_{ii}^2 \tau_{i2} \tau_{j2} \tau_{if} \tau_{jf}, & \cdots, & \sum_{i,j=1}^{n} C_{ij} A_{ii}^2 \tau_{if}^2 \tau_{jf}^2 + \beta \end{bmatrix}_{f \times f} \begin{bmatrix} w_{11} \\ w_{22} \\ \vdots \\ w_{ff} \end{bmatrix}_{f \times 1} = \begin{bmatrix} \sum_{i,j=1}^{n} C_{ij} R_{ij} A_{ii} \tau_{i1} \tau_{j1} \\ \sum_{i,j=1}^{n} C_{ij} R_{ij} A_{ii} \tau_{i2} \tau_{j2} \\ \vdots \\ \sum_{i,j=1}^{n} C_{ij} R_{ij} A_{ii} \tau_{if} \tau_{jf} \end{bmatrix}_{f \times 1}. \tag{11}$$

Similarly, the analytical solution of A can be obtained from

$$\frac{\partial L}{\partial A} = 0. \tag{12}$$

The result for A_{ii} is:

$$A_{ii} = \frac{\sum_{j=1}^{n} (TWT^t)_{ij} R_{ij}}{\sum_{j=1}^{n} C_{ij} (TWT^t)_{ij}^2}. \tag{13}$$

We update C based on the residual matrix Δ in every iteration. For a matrix $C' \in \mathbb{R}^{n \times n}$, we choose the k nearest neighbours for each cell. If cell j is one of the k nearest neighbours of cell i, $C'_{ij} = 1$; otherwise $C'_{ij} = 0$. Let ϕ_i denote the k nearest neighbours of cell i. The elements of the row vector C_i are initialized as follows:

$$C_{ij} = \mathbb{I}(j \in \phi_i) \quad j = 1, 2, \cdots, n. \tag{14}$$

Convergence of objective function

This study employs a specific condition to determine the convergence of the model. The convergence is measured by the difference between the current and previous losses, which must be less than 10^{-3} in absolute value. This condition serves as a threshold to determine whether the model has achieved sufficient convergence for this study. This condition was selected based on the trade-off between computational efficiency and model accuracy. A smaller threshold value would require more iterations to achieve convergence, increasing computational time. Conversely, a larger threshold could lead to premature termination of the model, resulting in suboptimal performance. Therefore, the value of 10^{-3} was selected as a compromise between these considerations. Additionally, this threshold is widely used in various machine learning studies, including those that involve deep neural networks and is effective in ensuring model convergence.

Formation of club hierarchy

Given a tree Υ of the sparse graph G_s constructed from the residual matrix Δ, if a leaf node $t \in \Upsilon$ can be divided into two child nodes μ and $\overline{\mu}$, then the T-cell clones V can be expressed as the union of $V(\mu)$ and $V(\overline{\mu})$, and $V(\mu) \cap V(\overline{\mu}) = \emptyset$. The volume of a node $\mu \in \Upsilon$ can be defined as the sum of degrees of all clones in $V(\mu)$. The structure entropy of μ can be expressed as

$$S^{\Upsilon}(G_s;\mu) = -\frac{g(\mu)}{vol(G)} log_2 \frac{vol(\mu)}{vol(t)}, \tag{15}$$

where $g(\mu)$ represents the total weight of edges from clones in $V(\mu)$ to $V - V(\mu)$.

Assuming that the new tree is denoted as Υ', the goal for finding the optimal bi-partition of leaf t is to maximize the structure entropy change from Υ to Υ'. This can be expressed as:

$$\begin{aligned} \text{maximize } \delta(t;\mu,\overline{\mu}) = & \sum_{u \in V(t)} S^{\Upsilon}(G_s;u) \\ & - \left[S^{\upsilon'}(G_s;\mu) + \sum_{u \in V(\mu)} S^{\Upsilon'}(G_s;u) \right] \\ & - \left[S^{\upsilon'}(G_s;\overline{\mu}) + \sum_{u \in V(\overline{\mu})} S^{\overline{\Upsilon}'}(G_s;u) \right] \\ = & \left[vol(\mu) \log_2 \frac{vol(t)}{vol(\mu)} + vol(\overline{\mu}) \log_2 \frac{vol(t)}{vol(\overline{\mu})} \right] \\ & - \left[g(\mu) \log_2 \frac{vol(t)}{vol(\mu)} + g(\overline{\mu}) \log_2 \frac{vol(t)}{vol(\overline{\mu})} \right]. \end{aligned} \tag{16}$$

To indicate what node the clone u belongs to, a vector $\mathbf{x} = \left[x_1, \ldots, x_{|V(t)|}\right]^t$ is introduced, where $|V(t)|$ denotes the number of clones inside leaf t. This can be expressed as

$$\mathbf{x_i} = \begin{cases} 1, & \text{if } u_i \in V(\mu), \\ 0, & \text{if } u_i \in V(\overline{\mu}). \end{cases} \tag{17}$$

Let L and D denote the Laplacian matrix and the degree matrix of G_s, respectively. The quantities $vol(\mu)$, $vol(\overline{\mu})$, $g(\mu)$, and $g(\overline{\mu})$ are defined as

$$\begin{aligned} vol(\mu) &= \mathbf{x}^t D \mathbf{x}, \\ vol(\overline{\mu}) &= (\mathbf{1} - \mathbf{x}^t) D (\mathbf{1} - \mathbf{x}), \\ g(\mu) &= \mathbf{x}^t L \mathbf{x}, \\ g(\overline{\mu}) &= (\mathbf{1} - \mathbf{x})^t L (\mathbf{1} - \mathbf{x}). \end{aligned} \tag{18}$$

When G_s is a regular graph, and the sum of $\mathbf{x}$ is restricted to $\frac{|V(t)|}{2}$, the maximization problem is reduced to a minimization problem:

$$\begin{aligned} \text{maximize } \delta(t;\mu,\overline{\mu}) &= \text{minimize } \Pi(t;\mu,\overline{\mu}) \\ &= \text{minimize } \frac{\mathbf{x}^t L \mathbf{x} + (\mathbf{1} - \mathbf{x})^t L (\mathbf{1} - \mathbf{x})}{\mathbf{x}^t D \mathbf{x}} \\ &= \text{minimize } \frac{\mathbf{x}^t L \mathbf{x}}{\mathbf{x}^t D \mathbf{x}} \\ \text{subject to } \mathbf{x^t x} &= \frac{|V(t)|}{2}, \\ vol(\mu) &= \frac{1}{2} vol(t), \\ vol(\overline{\mu}) &= \frac{1}{2} vol(t). \end{aligned} \tag{19}$$

Let L' denote the normalized Laplacian matrix $D^{-\frac{1}{2}} L D^{-\frac{1}{2}}$ and $\mathbf{y}$ denote $\sqrt{\frac{|V(t)|}{2}}\mathbf{x}$. The problem can be transferred to

$$\text{minimize } \Pi(t;\mu,\overline{\mu}) = \frac{\mathbf{y}^t L' \mathbf{y}}{\mathbf{y^t y}}. \tag{20}$$

We further relax $\mathbf{y} \in \mathbb{R}^{|V(t)|}$ to the continuous space. Assuming $\lambda = \frac{\mathbf{y}^t L' \mathbf{y}}{\mathbf{y^t y}}$, we have,

$$L'\mathbf{y} = \lambda \mathbf{y}. \tag{21}$$

According to the attribute of the normalized Laplacian matrix, $(0, \mathbf{1})$ should be the smallest eigenvalue and the corresponding eigenvector for the above equation. However, it contradicts the $\mathbf{y}$ definition. Therefore, the solution of $\mathbf{y}$ is the Fiedler vector of

L'. Finally, we can partition the leaf of Υ divisively until the leaf node contains only two clones or the structure entropy change δ_s is less than the threshold value ζ.

Setting of TCRclub Hyperparameters

On the dataset with over 400 verified pMHC specificity (Francis et al., 2021), we explored various settings for the following hyperparameters: the nearest neighbour parameter k, the L_2-regularized parameter β, the top m results yielding the smallest objective function to achieve a consensus clustering result, and the threshold value ζ for individual results as well as for the consensus result. We presented the average clustering purity and coverage of the dataset in Appendix Figure S33. After careful consideration of the results and balancing the trade-off between clustering purity and coverage, we have determined the default settings outlined in the main text for TCRclub.

Extension of TCRclub to additional contexts

Extreme clonal expansion does not affect the clustering results of TCRclub.

We investigated whether clustering results would differ under conditions of extreme clonal expansion compared to scenarios without such expansion. To simulate extreme clonal expansion, we artificially increased the number of cells within a clonotype. We tested $X \sim Poisson(0.1)$, $X \sim Poisson(0.5)$, and $X \sim Poisson(0.9)$ to simulate dropouts in the Gaussian noise. The expression profiles of expanded cells were simulated by adding Gaussian noise to the original clonotype's expression. We found no difference in clustering results. The results for $X \sim Poisson(0.5)$ are shown in the Appendix Figure S4.

TCRclub extends the view of partition-based graph abstraction in drawing the topology of data

Partition-based graph abstraction (PAGA) (Wolf et al., 2019) is a trajectory-inference algorithm which provides a graph-map of data. It uses gene expression data for computing neighbourhood relations among cells. However, in this study, we utilized the residual-distance matrix generated by TCRclub to construct a kNN graph. Revisiting the TCRclub algorithm, a stable residual-distance matrix is obtained once the objective function reaches convergence. Each element within this matrix signifies the distance between clones, taking into account both scRNA expression and scTCR sequences. Given that PAGA requires cell distances, we expanded the residual-distance matrix from a clone-clone basis to a cell-cell basis by selecting a representative cell from each clone in every sample. The gene expression distances between the representative cell and the other cells within the clone are most closely aligned with the average pair-wise gene expression distances amongst the clones. The neighbourhood of the representative cell can be represented by other representative cells from different clones, and the neighbourhood of the cells within the same clone mirrors that of the representative cell of the clone. As depicted in Appendix Figure S26A, following the construction of a new graph based on the residual-distance from TCRclub, we employed PAGA to connect the partitions (such as clusters, celltypes) within the graph.

We integrated pre- and post-treatment CD8 T cell samples from a non-small cell lung cancer patient (P1) (Liu et al., 2022) using Seurat(v4.3.0). Subsequently, TCRclub was applied to the integrated data to generate a residual-distance matrix. PAGA was then employed on both the default kNN-like graph based on gene expressions (Appendix Figure S26B) and the new kNN-like graph based on TCRclub (Appendix Figure S26C), with cell type as the partition. The connectivity observed in the TCRclub-based graph (Appendix Figure S26C) appeared more biologically plausible compared to the gene expression-based graph (Appendix Figure S26B), capturing relationships between proliferative, terminal exhausted, and non-exhausted T cells. Given the smaller size of the CD8 dataset, we also integrated pre- and post-treatment CD4 T cell samples from the same patient and applied TCRclub. Due to the large number of clubs, we utilized TCRclub's subpopulation layer to partition the graph (Appendix Figure S26D-F). The subpopulation layer is the upper layer of the club layer, and the number of subpopulations is much less than the number of clubs because of the tree-like structure. Comparisons were made using gene expression distances (Appendix Figure S26D), TCR embedding distances (Appendix Figure S26E), and TCRclub's residual-distance matrix (Appendix Figure S26F). Interestingly, the graph based on TCRclub's residual-distance matrix exhibited a balance between the gene expression-based and TCR embedding-based graphs. In the gene expression-based graph, weights between subpopulations 8 and 9 (red line) and between subpopulations 7 and 9 (blue line) were equal (Appendix Figure S26D). However, in the TCR embedding-based graph (Appendix Figure S26E), these weights were lower than those in Appendix Figure S26D. Conversely, the graph based on TCRclub's residual-distance matrix (Appendix Figure S26F) considered the weights between subpopulations 7 and 9, and 8 and 9, akin to the TCR embedding-based graph. Furthermore, we partitioned the kNN graph using celltypes based on gene expression distances (Appendix Figure S26G), TCR embedding distances (Appendix Figure S26H), and TCRclub's residual-distance matrix (Appendix Figure S26I), yielding consistent results as with subpopulations.

Overall, we discussed the potential extension of TCRclub to cell trajectory analysis. and we found that TCRclub enriches the perspective of gene expression analysis by integrating TCR sequences. While TCRclub primarily focuses on T-cell clustering, we anticipate its broader application in studying cell trajectories.

TCRclub reveals functionally-similar T cells are dispersed in tumor microenvironment of brain metastases

In addition to its application to the matched scRNA and scTCR data discussed in the main text, we extended the use of TCRclub to explore spatial transcriptomic (ST) data. Specifically, We utilized TCRclub, with default settings, to analyze a dataset (Hudson and Sudmeier, 2022) comprising ST data of brain metastases, along with matched scRNA-seq and scTCR-seq data specifically focused on T cells within the brain metastases. Because the dataset only contains the scRNA-seq data of T cells, in order to try our best to restore a tumor microenvironment (TME), we integrated the scRNA expressions with the scRNA expressions of multiple cell types from another brain metastases dataset (Wang et al., 2019) through Seurat (v4.3.0). Subsequently, we performed cell annotation on the integrated expression data utilizing singleR (v2.0.0). Leveraging the cell annotation, we conducted deconvolution analysis and mapped the cells in the integrated data to their corresponding spatial spots using CytoSPACE (Vahid et al., 2023).

Annotated by the work of Sudmeier et al. (Sudmeier et al., 2022), the tissue was divided into four primary areas (Appendix Figure S27A). As various cell types are present in each spot, we assign the type of a spot as the type of the largest group of cells in the spot (Appendix Figure S28A). To examine the cell-cell interactions that regulate the immune response, we used the neighbourhood enrichment test (Palla et al., 2022; Schapiro et al., 2017) for the spots by Squidpy (v1.2.3). The neighbourhood enrichment test is an unbiased and systematic approach to examining the interactions between non-randomly distributed objects within the tissue. It calculates the z-score to quantify the strength of the interaction. Higher neighbourhood enrichment values indicate a greater likelihood of interaction between the cells. Our findings revealed that T cells only displayed high enrichment values with T cells (Appendix Figure S27B), suggesting a tendency for T cells to occur in isolation rather than interacting with other cell types in TME. The limited interaction of T cells with other cell types, particularly tumor cells, suggests a potential immunosuppressive microenvironment (Cess and Finley, 2020).

We aimed to investigate potential differences in club distribution between the immune area and the tumor area. First, we identified the T-cell clubs on the matched scRNA-seq and scTCR-seq data from the dataset (Hudson and Sudmeier, 2022) by TCRclub. Then, we analyzed the T-cell clubs associated with the immune area and the tumor area. Specifically, we focused on the clubs that contained T cells from the inflammatory area and ranked them based on the number of T cells originating from this region. From this ranked list, we extracted the ten largest T-cell clubs in the inflammatory area. Similarly, we applied the same workflow to the clubs containing T cells from tumor area, ranking the clubs according to the number of T cells from the tumor area and extracting the ten largest T-cell clubs. We observed six clubs consistently among the largest in the inflammatory and tumor tissues (Appendix Figure S27C). The club that occupies a dominant position in inflammation may not necessarily still have the same status in tumor tissues, and may even disappear. The result suggests that although the TME can be infiltered by some T cells, it may not be as conducive to T cell proliferation and recruitment as the inflammation tissue, indicating a relative lack of immune surveillance and lower efficacy of the anti-tumor immune response in the TME (Wang et al., 2022).

To further investigate the spatial characteristics of the T-cell clubs, we analyzed the spatial distribution of the five largest clubs in the inflammation area. We assessed their spatial patterns using Ripley's L function (Palla et al., 2022). Ripley's L function characterises whether cell clusters exhibit random, dispersed, or clustered patterns. Interestingly, the curves representing the five largest clubs within the inflammation were consistently below the expected curve of a random spatial distribution (Appendix Figure S27D), suggesting that T cells with similar functions are more dispersed in the tissue. This observation may be attributed to the heterogeneous composition of the TME, which fails to provide concentrated signals that attract functionally similar T cells to specific locations (Zhang et al., 2020; Kohli et al., 2022). However, we observed that the five largest clubs tended to cluster near the tumor-inflammation adjacent area, primarily located in the bottom right corner of the tissue (Appendix Figure S27E). To explore the reasons behind this T cell concentration, we conducted an analysis of differentially expressed genes (DEGs) across the inflammation, tumor, and adjacent regions near the concentrated area (Appendix Figure S28B, S27F). The expressions of the DEGs in the adjacent region, near the concentrated area, are illustrated in Appendix Figure S27G. Among these DEGs, HLA, CD74, TNC, and TMSB4X are known to regulate antigen presentation and cell migration, which suggests the occurrence of frequent antigen-presenting and recognition activities that attract T cells to this specific location (Appendix Figure S27G).

Overall, in the context of brain metastases, TCRclub offered a detailed view that T cells of the same club are dispersed in the spatial distribution, indicating the inefficiency in their collective anti-tumor response due to immune system disruptions. However, a limitation is the scarcity of available data suitable for applying TCRclub in ST. We anticipate further validation with ample and comprehensive scRNA-seq data, ST data for tumor cells, and scTCR-seq data for T cells within tumors.

References

Breiman, L. (1996). Bagging predictors. *Machine learning*, 24:123–140.

Cess, C. G. and Finley, S. D. (2020). Multi-scale modeling of macrophage—t cell interactions within the tumor microenvironment. *PLoS computational biology*, 16(12):e1008519.

Dudoit, S. and Fridlyand, J. (2003). Bagging to improve the accuracy of a clustering procedure. *Bioinformatics*, 19(9):1090–1099.

Francis, J. M., Leistritz-Edwards, D., Dunn, A., Tarr, C., Lehman, J., Dempsey, C., Hamel, A., Rayon, V., Liu, G., Wang, Y., et al. (2021). Allelic variation in class i hla determines cd8+ t cell repertoire shape and cross-reactive memory responses to sars-cov-2. *Science immunology*, 7(67):eabk3070.

Hudson, W. H. and Sudmeier, L. J. (2022). Localization of t cell clonotypes using the visium spatial transcriptomics platform. *STAR protocols*, 3(2):101391.

Kingma, D. P. and Welling, M. (2013). Auto-encoding variational bayes. *arXiv preprint arXiv:1312.6114*.

Kohli, K., Pillarisetty, V. G., and Kim, T. S. (2022). Key chemokines direct migration of immune cells in solid tumors. *Cancer gene therapy*, 29(1):10–21.

Lee, K. and Stöger, D. (2023). Randomly initialized alternating least squares: Fast convergence for matrix sensing. *SIAM Journal on Mathematics of Data Science*, 5(3):774–799.

Liu, B., Hu, X., Feng, K., Gao, R., Xue, Z., Zhang, S., Zhang, Y., Corse, E., Hu, Y., Han, W., et al. (2022). Temporal single-cell tracing reveals clonal revival and expansion of precursor exhausted t cells during anti-pd-1 therapy in lung cancer. *Nature cancer*, 3(1):108–121.

Luo, J., Wang, X., Zou, Y., Chen, L., Liu, W., Zhang, W., and Li, S. C. (2023). Quantitative annotations of t-cell repertoire specificity. *Briefings in Bioinformatics*, 24(3):bbad175.

Palla, G., Spitzer, H., Klein, M., Fischer, D., Schaar, A. C., Kuemmerle, L. B., Rybakov, S., Ibarra, I. L., Holmberg, O., Virshup, I., et al. (2022). Squidpy: a scalable framework for spatial omics analysis. *Nature methods*, 19(2):171–178.

Schapiro, D., Jackson, H. W., Raghuraman, S., Fischer, J. R., Zanotelli, V. R., Schulz, D., Giesen, C., Catena, R., Varga, Z., and Bodenmiller, B. (2017). histocat: analysis of cell phenotypes and interactions in multiplex image cytometry data. *Nature methods*, 14(9):873–876.

Shahriari, M., Ramler, R., and Fischer, L. (2022). How do deep-learning framework versions affect the reproducibility of neural network models? *Machine Learning and Knowledge Extraction*, 4(4):888–911.

Sudmeier, L. J., Hoang, K. B., Nduom, E. K., Wieland, A., Neill, S. G., Schniederjan, M. J., Ramalingam, S. S., Olson, J. J., Ahmed, R., and Hudson, W. H. (2022). Distinct phenotypic states and spatial distribution of cd8+ t cell clonotypes in human brain metastases. *Cell Reports Medicine*, 3(5):100620.

Swirszcz, G., Czarnecki, W. M., and Pascanu, R. (2016). Local minima in training of neural networks. *arXiv preprint arXiv:1611.06310*.

Vahid, M. R., Brown, E. L., Steen, C. B., Zhang, W., Jeon, H. S., Kang, M., Gentles, A. J., and Newman, A. M. (2023). High-resolution alignment of single-cell and spatial transcriptomes with cytospace. *Nature Biotechnology*, pages 1–6.

Vita, R., Mahajan, S., Overton, J. A., Dhanda, S. K., Martini, S., Cantrell, J. R., Wheeler, D. K., Sette, A., and Peters, B. (2019). The immune epitope database (iedb): 2018 update. *Nucleic acids research*, 47(D1):D339–D343.

Wang, D.-R., Wu, X.-L., and Sun, Y.-L. (2022). Therapeutic targets and biomarkers of tumor immunotherapy: response versus non-response. *Signal Transduction and Targeted Therapy*, 7(1):331.

Wang, L., Dai, J., Han, R.-R., Dong, L., Feng, D., Zhu, G., Guo, W., Wang, Y., Chao, M., Jin, M.-z., et al. (2019). Single-cell map of diverse immune phenotypes in the metastatic brain tumor microenvironment of non small cell lung cancer. *bioRxiv*, page 890517.

Wen, Y., Zhang, K., Li, Z., and Qiao, Y. (2016). A discriminative feature learning approach for deep face recognition. In *Computer Vision–ECCV 2016: 14th European Conference, Amsterdam, The Netherlands, October 11–14, 2016, Proceedings, Part VII 14*, pages 499–515. Springer.

Wolf, F. A., Hamey, F. K., Plass, M., Solana, J., Dahlin, J. S., Göttgens, B., Rajewsky, N., Simon, L., and Theis, F. J. (2019). Paga: graph abstraction reconciles clustering with trajectory inference through a topology preserving map of single cells. *Genome biology*, 20:1–9.

Zhang, Y., Guan, X.-y., and Jiang, P. (2020). Cytokine and chemokine signals of t-cell exclusion in tumors. *Frontiers in immunology*, 11:594609.
